# Evaluation of electrocardiogram: numerical vs. image data for emotion recognition system

**DOI:** 10.12688/f1000research.73255.1

**Published:** 2021-11-04

**Authors:** Sharifah Noor Masidayu Sayed Ismail, Nor Azlina Ab. Aziz, Siti Zainab Ibrahim, Sophan Wahyudi Nawawi, Salem Alelyani, Mohamed Mohana, Lee Chia Chun

**Affiliations:** 1 Faculty of Information Science & Technology, Multimedia University, Bukit Beruang,, Melaka, 75450, Malaysia; 2 Faculty of Engineering, Multimedia University, Bukit Beruang, Melaka, 75450, Malaysia; 3 School of Electrical Engineering, Faculty of Engineering, Universiti Teknologi Malaysia, Skudai, Johor Bahru, 81310, Malaysia; 4 Center for Artificial Intelligence, King Khalid University, Abha, 61421, Saudi Arabia; 5 College of Computer Science, King Khalid University, Abha, 61421, Saudi Arabia; 6 Hexon Data Sdn Bhd, Kuala Lumpur, 59200, Malaysia

**Keywords:** Emotion recognition, electrocardiogram, numerical ECG, image ECG, DREAMER

## Abstract

**Background: **The electrocardiogram (ECG) is a physiological signal used to diagnose and monitor cardiovascular disease, usually using ECG wave images. Numerous studies have proven that ECG can be used to detect human emotions using numerical data; however, ECG is typically captured as a wave image rather than as a numerical data. There is still no consensus on the effect of the ECG input format (either as an image or a numerical value) on the accuracy of the emotion recognition system (ERS). The ERS using ECG images is still inadequately studied. Therefore, this study compared ERS performance using ECG image and ECG numerical data to determine the effect of the ECG input format on the ERS.

**Methods: **This study employed the DREAMER dataset, which contains 23 ECG recordings obtained during audio-visual emotional elicitation. Numerical data was converted to ECG images for the comparison. Numerous approaches were used to obtain ECG features. The Augsburg BioSignal Toolbox (AUBT) and the Toolbox for Emotional feature extraction from Physiological signals (TEAP) extracted features from numerical data. Meanwhile, features were extracted from image data using Oriented FAST and rotated BRIEF (ORB), Scale Invariant Feature Transform (SIFT), KAZE, Accelerated-KAZE (AKAZE), Binary Robust Invariant Scalable Keypoints (BRISK), and Histogram of Oriented Gradients (HOG). Dimension reduction was accomplished using linear discriminant analysis (LDA), and valence and arousal were classified using the Support Vector Machine (SVM).

**Results: **The experimental results indicated that numerical data achieved arousal and valence accuracy of 69% and 79%, respectively, which was greater than those of image data. For ECG images, the highest accuracy for arousal was 58% percent; meanwhile, the valence was 63%.

**Conclusions: **The finding showed that numerical data provided better accuracy for ERS. However, ECG image data which shows positive potential and can be considered as an input modality for the ERS.

## Introduction

Medical professionals have been actively using electrocardiogram (ECG) wave images as a tool for monitoring ^1^^,^^2^ and diagnosing ^3^^–^^6^ cardiovascular diseases, such as heart attacks, dysrhythmia, and pericarditis, with some reported accuracy of more than 99% in the past decade. Besides monitoring and diagnosing health-related diseases, many studies have proven that human emotions can also be identified using ECG in the form of numerical data. ^7^^–^^10^

The effects of using different types of ECG inputs to recognise emotions by the emotion recognition system (ERS) have yet to be closely studied. In addition, there is no consensus on whether or not the type of ECG input format affects the emotion classification accuracy by the ERS. Most researchers have focused on recognising emotions using ECG numerical data instead of using EGC wave images. To date, research on the use of ECG wave images in identifying emotions is still absent. Therefore, to address this gap, the objective of this study is to compare emotion classification performance using ECG image and ECG numerical data to determine the effect of the ECG input format on the ERS.

### Emotion model

**Figure 1.**
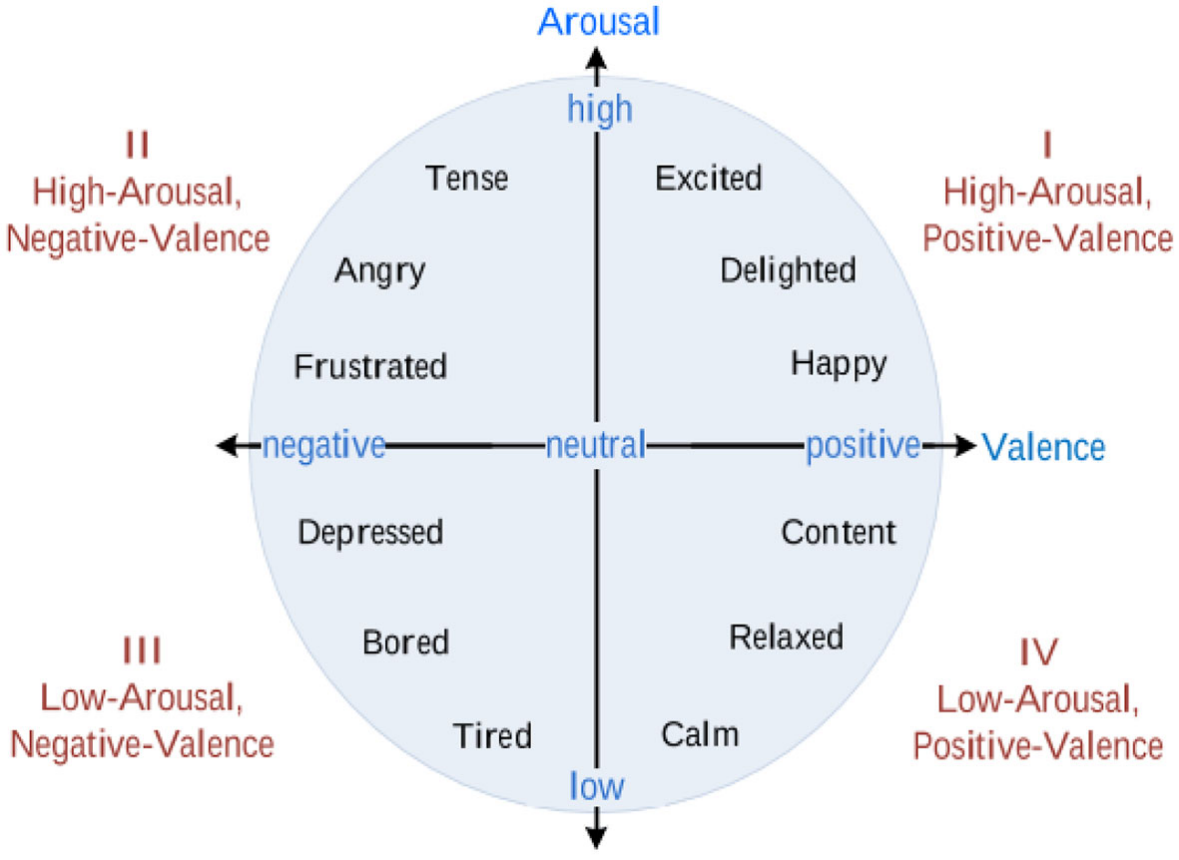
The valence arousal scale. ^14^

Emotions can be seen in two different models put forward by Paul Ekman ^11^ and James Russell, ^12^ namely, the discrete emotion and dimensional emotion models. Ekman, a psychologist, suggested six basic emotions: happiness, sadness, anger, fear, disgust, and surprise. On the other hand, Russell presented a two-dimensional scale of emotions consisting of valence and arousal ( Figure 1). Valence refers to positive or negative feelings, and arousal indicates the intensity of the feeling, either high or low. This study used the latter emotion model to classify the subject’s emotions, because 1) the work presented in ^13^ used the same emotion model; hence, allowing benchmarking of the performance, and 2) simplicity of binary classification.

### Electrocardiogram & Emotion

An electrocardiogram is used to measure electrical activity in the human heart by attaching electrodes to the human body. The standard is a 12-lead ECG system. However, today ECG devices have evolved from bulky nonportable devices to wearable portable devices. The accuracy of the signal by portable devices is comparable to conventional medical devices. This suggests that researchers can use wearable ECG devices for purposes similar to conventional devices, including for studying human emotions. However, most of these devices store the ECG as images instead of raw numerical data.

**Figure 2.**
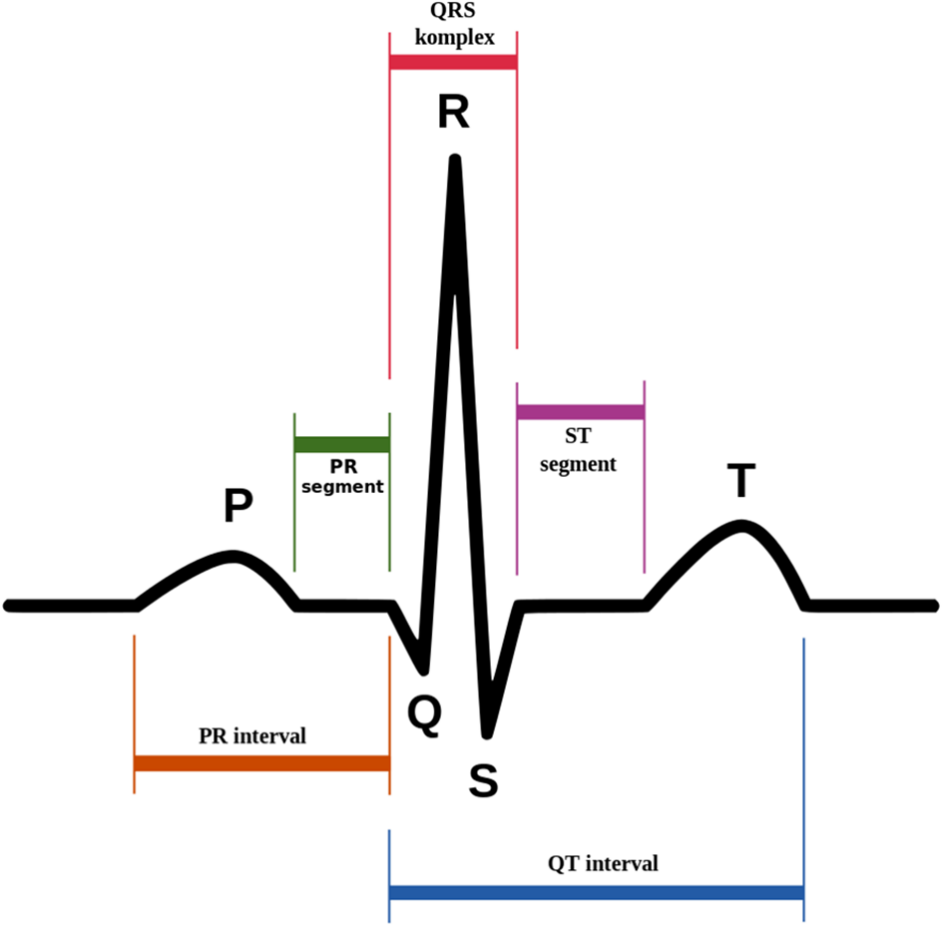
The P wave, QRS complex, and T wave in the standard electrocardiogram (ECG). ^17^

The ECG signals have P, Q, R, S, and T waves ( Figure 2). Emotional states are associated with autonomic nervous system's (ANS) physiological responses. ^15^ Different emotions influence human heart activities differently; these influences may be hidden in the ECG wave. ^16^ These responses can be detected through ECG by monitoring the main features of ECG, namely, heart rate (HR) and heart rate variability (HRV).

This study used ECG numerical data obtained from the multimodal database called the DREAMER dataset. ^13^ In the dataset, electroencephalogram (EEG) and ECG signals were recorded from 23 participants during an emotion elicitation session. The emotions were elicited by using 18 audio-visual stimulations. Valence and arousal emotion model was used to classify the elicited emotions. This paper only focuses on the use of ECG. We employed the Augsburg BioSignal Toolbox (AUBT) ^18^ and the Toolbox for Emotional feature extraction from Physiological signals (TEAP) ^19^ to facilitate feature extraction from the ECG numerical data. Then, linear discriminant analysis (LDA) was applied to reduce the dimension of the extracted ECG numerical features.

Since the DREAMER dataset only has numerical data, the data must be converted into the corresponding ECG wave images for comparison purposes. Six different feature extractors, namely, Oriented FAST and rotated BRIEF (ORB), Scale Invariant Feature Transform (SIFT), KAZE, Accelerated-KAZE (AKAZE), Binary Robust Invariant Scalable Keypoints (BRISK), and Histogram of Oriented Gradients (HOG), were applied to the ECG wave images to detect and extract features. The Support Vector Machine (SVM) was used to classify the valence and arousal of both ECG numerical features and ECG image features.

In the following section, an overview of related works on the ERS is presented. We then describe the selected dataset and explain the proposed methods in detail. This is followed by the results and a discussion and conclusions section.

## Related works

Researchers in the emotion recognition field have been proposing multiple approaches using electrocardiogram signals. For instance, Minhad, Ali, and Reaz ^20^ used ECG numerical data to classify emotions of happiness and anger. They achieved 83.33% accuracy using the SVM classification method. Besides, Tivatansakul and Ohkura ^21^ used ECG numerical data from the AUBT dataset to detect emotions for the emotional healthcare system. K-Nearest Neighbour (KNN) successfully classified three emotions (joy, anger, and sadness) with an accuracy 85.75%, 82.75%, and 95.25%, respectively.

Katsigiannis and Ramzan suggested that ERS should use low-cost and off-the-shelf devices to collect ECG signals based on numerical format. ^13^ AUBT and Biosig Toolbox were used to extract the signal features. Classification using SVM with a radial basis function kernel successfully achieved 62.37% for valence and arousal. The MPED database for ERS was proposed by Song *et al.* ^22^ using ECG numerical data to recognise discrete emotions (joy, humour, disgust, anger, fear, sadness, and neutrality). Attention Long Short-Term Memory (A-LSTM) was used as a feature extractor to extract the frequency and time-domain features from the physiological signal. The A-LSTM was used as a classifier along with SVM, KNN, and Long Short-Term Memory (LSTM). Averagely, A-LSTM achieved better results of 40% to 55% compared to those of other classifiers.

Just as with the widespread use of numerical ECG in human emotion studies, ECG images are also widely used to identify cardiovascular-related diseases. For example, Hao *et al.* ^23^ used ECG images to detect and classify myocardial infarction (MI). MI is a disease caused by severe cardiovascular obstruction that leads to irreversible injury or even death. KNN and SVM were used in this study and achieved 89.84% and 92.19%, respectively. Besides, Mandal, Mondal, and Roy ^24^ used ECG images to detect ventricular arrhythmia (VA), such as ventricular tachycardia (VT) and ventricular fibrillation (VF) in infants and children. SVM, KNN, and random forest (RF) were used in this study and successfully achieved 93.11%, 92.56%, and 95.36%, respectively.

Although much research has been conducted using ECG for ERS, most of them focused mainly on numerical data analysis instead of ECG wave images. However, systems based on ECG images have achieved excellent results in detecting cardiovascular-related diseases. As mentioned before, it remains uncertain whether the ECG input format, numerical value or wave image, affects the emotional classification accuracy in the ERS. Therefore, it is essential to explore the ERS using different input formats of ECG to address this knowledge gap.

## Methods

### Proposed method

**Figure 3.**
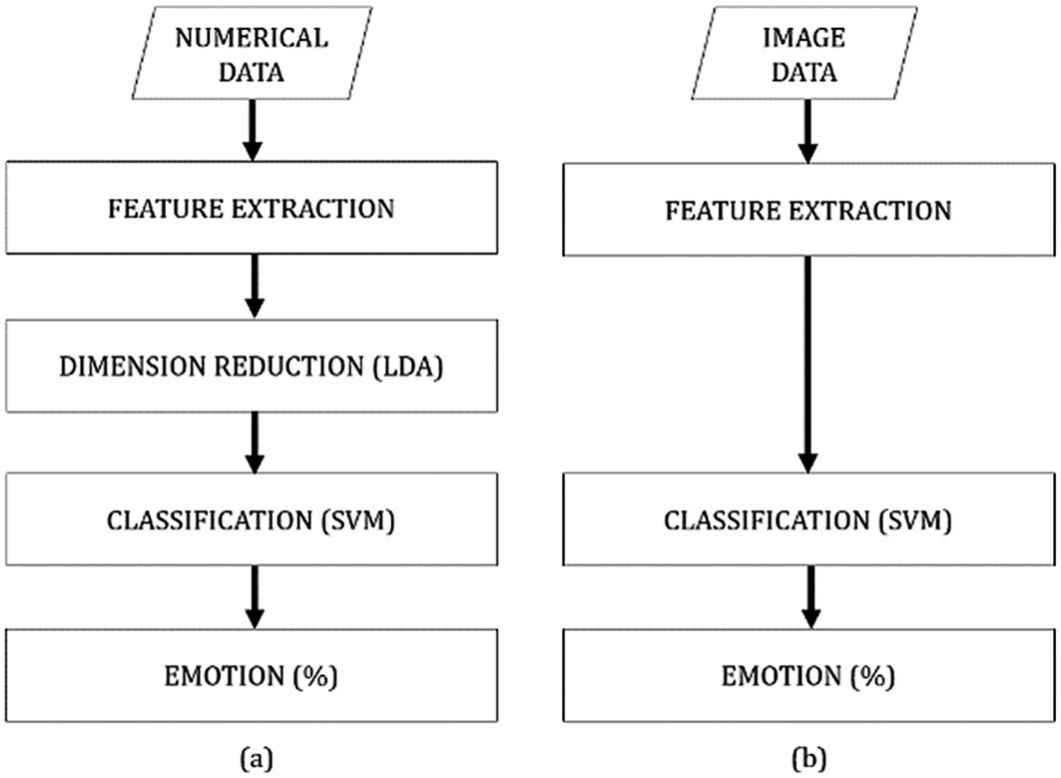
Structure of emotion recognition system: (a) numerical data and (b) image data.

The proposed method consists of three stages: feature extraction, feature dimension reduction, and emotion classification. The data of the present study were obtained in the experiment described in the original study. ^13^ The current study began in September 2020. Matlab version 9.7 was utilized for data conversion and feature extraction, whereas Python version 3.8.5 was used for feature dimension reduction (numerical) and classification. The overall structure of the proposed method is illustrated in Figure 3. The analysis code used in this study is available from GitHub and archived with Zenodo. ^41^

### The dataset (DREAMER)

We built our ERS on a publicly accessible database consisting of ECG signals recorded from 23 participants during emotion elicitation by audio-visual stimuli. The ECG was recorded using the SHIMMER ECG sensor at 256 Hz. Nine emotions: calmness, surprise, amusement, fear, excitement, disgust, happiness, anger, and sadness were elicited using 18 video excerpts.

The total data amount is 414 data (23 subjects x 18 videos). As previously mentioned, this work is only interested in ECG signals; hence, EEG signals are not included in this study. Additionally, we did not use the dominance rating score, since Russell’s two-dimensional emotional model is adapted here to classify emotions. The summary of the DREAMER dataset is tabulated in Table 1.

**Table 1.** The summary of the dataset.

**No of subject**	23
**No of videos**	18 audio-visual stimuli
**Type of stimuli**	Audio-video
**Used Signal (Hz)**	ECG (256)
**Rating scales**	Valence, Arousal
**Rating values**	1 – 5

### Experimental setup

1) ECG wave image

**Figure 4.**
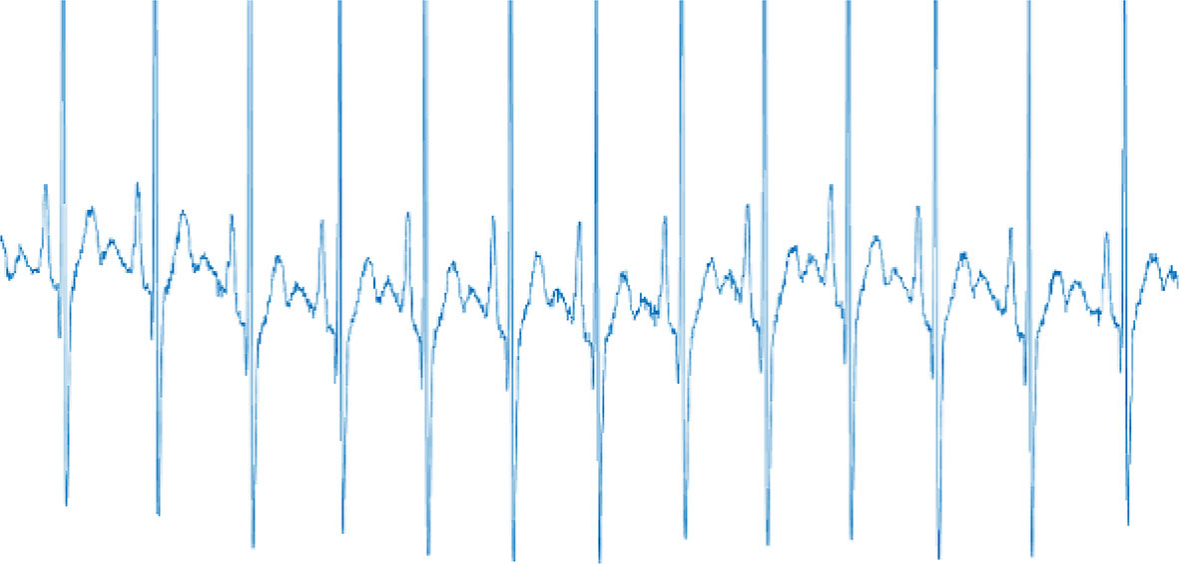
The electrocardiogram (ECG) wave image converted from ECG numerical data.

The ECG numerical data was converted into ECG wave images preceding the analysis of ECG wave images using MATLAB version 9.7 ( Figure 4). Using Python version 3.8.5, the converted ECG images were then resized to 60% of the original size to reduce the computational time. After resizing, the coloured images were converted into greyscale images. Then, binarization of the image using a threshold was done. Automatic image thresholding, Otsu’s method, ^25^ was used here. Otsu’s method ascertains the optimal threshold values from pixel values of 0 to 255 by calculating and evaluating their within-class variance. This method provides the best performance, as stated in. ^26^

**Figure 5.**
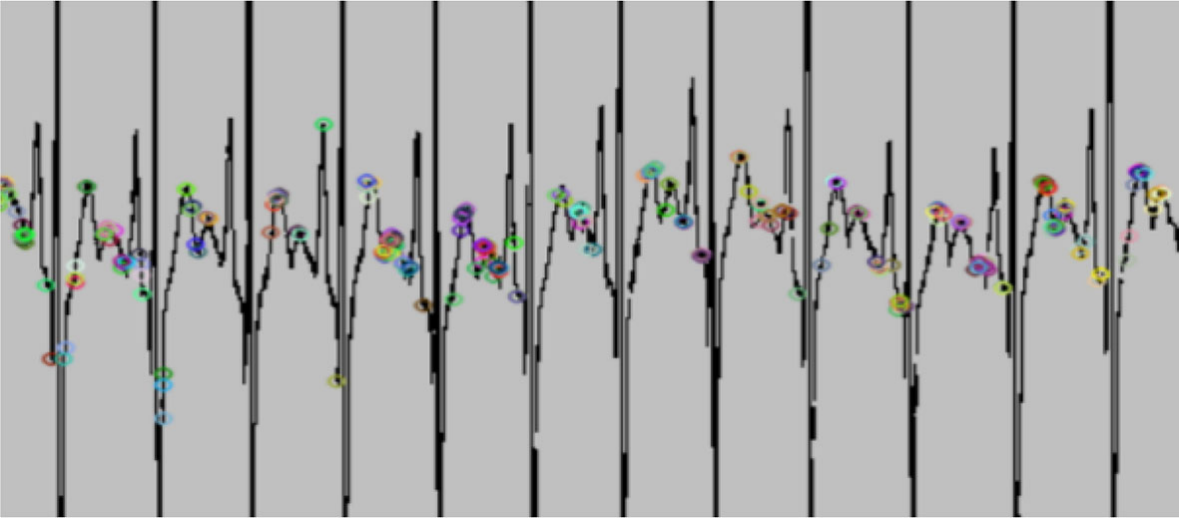
Oriented FAST and rotated BRIEF (ORB) is unable to detect the electrocardiogram (ECG) peaks due to the presence of noise.

In total, six different feature extractors were applied to extract features from processed ECG images. The feature extractors are as follows: ORB, ^27^ SIFT, ^28^ KAZE, ^29^ AKAZE, ^30^ BRISK, ^31^ and HOG. ^32^ All of them successfully extracted the ECG features, including the peaks, edges, and corners. However, some feature extractors, such as ORB and SIFT, failed to detect important features, particularly the R-peaks, due to the presence of mass noise, ^33^ which is believed to have affected emotional classification ( Figure 5). The extracted images were then given to the classifier (SVM) to classify valence and arousal. This whole process of feature extraction and classification was done using Python version 3.8.5.

### ECG numerical data

Numerical ECG data did not go through any pre-processing process as suggested in ^13^ due to being less susceptible to interference owing to their higher voltage amplitudes. Two open-source toolboxes, namely, Augsburg BioSignal Toolbox (AUBT) ^18^ and Toolbox for Emotional feature extraction from Physiological signals (TEAP), ^19^ were employed to facilitate feature extraction from the ECG signals. Both of them successfully extracted 81 features ( Table 2) and 16 features ( Table 3) from the ECG signals. The extracted features included heart rate variability (HRV), inter-beat interval (IBI), tachogram power, and statistical features such as mean, median, and standard deviation. The dimension of the features was reduced using linear discriminant analysis, one of the well-known feature reduction methods. ^34^ This process was performed to reduce the computational cost and to improve the separation of emotion classes. ^35^ The extracted features were then given to the classifier (SVM) to classify valence and arousal.

**Table 2.** Features extracted from Augsburg Bio-signal Toolbox (AUBT).

Features	Description
P, Q, R, S, T	P-, Q-, R-, S-, T-peaks (ECG)
HRV	Heart rate variability
Ampl	Amplitude Signal
Mean	Mean value
Median	Median value
Std	Standard deviation
Min	Minimum value
Max	Maximum value
SpecRange	Mean of the frequency spectrum in a given range

**Table 3.** Features extracted from Toolbox for Emotional feature extraction from Physiological signals (TEAP).

Features	Description
meanIBI	Mean inter-beat interval
HRV	Heart Rate Variability
MSE	Multiscale entropy at 5 levels
sp0001/0102/0203/0304	Spectral power 0-0.1Hz, 0.1-0.2Hz, 0.2-0.3Hz, 0.3-0.4Hz
energyRatio	Spectral energy ratio between f<0.08Hz/f>0.15Hz and f<5.0Hz
tachogram_LF/MF/HF	Spectral power in tachogram (HRV) for low, medium, and high frequencies.
tachogram_energy_ratio	Energy ratio for tachogram spectral content (MF/(LF+HF))

### Support vector machine

Classification was performed using SVM. The SVM works by separating the class data points and drawing a boundary called the hyperplane between them. Additionally, SVM has a low computational cost and shows excellent performance in classifying emotions, as reported in previous studies. ^13^^,^^36^^,^^37^ The data was then divided into training and test sets with a ratio of 80:20. The parameters for SVM were tuned using GridSearchCV. ^38^ As we had a small data size, we used 10-fold cross-validation to improve ERS performance. The emotions were determined as follows: high/low valence and high/low arousal, based on the participants’ self-assessment rating.

## Results

The experimental results for numerical data showed that the accuracy of arousal achieved using the TEAP feature extractor (69%) was higher than that of the AUBT feature (64%). However, the TEAP feature managed to obtain 67% valence accuracy, while the AUBT feature recorded up to 79%. These results are better than what were recorded in Ref. 11.

The classification results using ECG wave images recorded an arousal accuracy of 53% to 58%. The highest result was achieved by the SIFT feature, followed by ORB, HOG, KAZE, BRISK, and the AKAZE features. Meanwhile, the highest accuracy for valence was attained by the KAZE feature with 63%, followed by HOG, BRISK, AKAZE, SIFT, and lastly, ORB with 48%, the lowest among other features. The results of this study are presented in Table 4.

**Table 4.** Testing emotion classification accuracy for electrocardiogram (ECG) numerical data and ECG image.

Type of ECG	Feature extractor	Arousal accuracy	Valence accuracy
Image	ORB	0.57	0.48
	SIFT	0.58	0.51
	AKAZE	0.53	0.54
	BRISK	0.54	0.58
	HOG	0.57	0.60
	KAZE	0.54	0.63
Numerical data	TEAP	0.69	0.67
	AUBT	0.64	0.79
	AUBT *	0.62	0.62

* Accuracy of DREAMER reported in the original paper.

## Discussion & conclusions

Findings showed that numerical data provided better accuracy for ERS compared to ECG images. In addition, numerical data was easier to handle and process compared to image data. Moreover, the results obtained here using ECG numerical data were even better than those reported by DREAMER. ^13^ This is contributed by the additional processes in our proposed method, the feature reduction using LDA, which was not included in the DREAMER paper. LDA plays an essential role in improving the performance of the emotion recognition system. ^35^^,^^39^ On top of that, it is worth noting that the results obtained using ECG image data also showed positive potential and could be considered as an input modality for the ERS. The features extracted by KAZE provided 63% accuracy for valence, which is better than the original work in Ref. 11. Hence, ECG images are recommended for building ERS. ECG images are attractive as the format allows usage of many image-based methods such as image augmentation to increase the data size, the convolution neural networks (CNN), and application of transfer learning from models trained using large data.

However, some limitations were found throughout the study and needed to be addressed to achieve better emotion classification results. The first limitation is, as per suggestion by the DREAMER paper, we did not run the pre-process process to the signal leading to the presence of noise in the signal, both ECG data format, which have affected emotion classification, especially for image data. Therefore, the use of filtering and noise reduction in the pre-processing stage should be considered. The second limitation is the data size, which is too small for image learning and classification, leading to lower accuracy. ^40^ In the future, with a larger data size, researchers can consider deep learning techniques for emotion classification using ECG images as a primary modality.

To conclude, ECG numerical data provided a better performance of emotion classification. In addition, ECG image data that shows positive potential, thus it can be considered an input modality for the ERS in future studies.

## Data availability

### Source data

The DREAMER dataset was first presented here: https://doi.org/10.1109/JBHI.2017.2688239 and can be found on Zenodo. Access is restricted and users are required to apply. The decision whether to grant/deny access is solely under the responsibility of the record owner.

### Extended data

Analysis code available from: https://github.com/nr-isml/ECG-Numerical-Vs.-Image-Data-for-Emotion-Recognition-System

Archived analysis code as at time of publication: https://doi.org/10.5281/zenodo.5542739. ^41^

License: Data are available under the terms of the Creative Commons Zero “No rights reserved” data waiver (CC0 1.0 Public domain dedication).
