# Evaluation of electrocardiogram: numerical vs. image data for emotion recognition system

**DOI:** 10.12688/f1000research.73255.2

**Published:** 2022-05-30

**Authors:** Sharifah Noor Masidayu Sayed Ismail, Nor Azlina Ab. Aziz, Siti Zainab Ibrahim, Sophan Wahyudi Nawawi, Salem Alelyani, Mohamed Mohana, Lee Chia Chun

**Affiliations:** 1 Faculty of Information Science & Technology, Multimedia University, Bukit Beruang,, Melaka, 75450, Malaysia; 2 Faculty of Engineering, Multimedia University, Bukit Beruang, Melaka, 75450, Malaysia; 3 School of Electrical Engineering, Faculty of Engineering, Universiti Teknologi Malaysia, Skudai, Johor Bahru, 81310, Malaysia; 4 Center for Artificial Intelligence, King Khalid University, Abha, 61421, Saudi Arabia; 5 College of Computer Science, King Khalid University, Abha, 61421, Saudi Arabia; 6 Hexon Data Sdn Bhd, Kuala Lumpur, 59200, Malaysia

**Keywords:** Emotion recognition, electrocardiogram, numerical ECG, image ECG, DREAMER

## Abstract

**Background: **The electrocardiogram (ECG) is a physiological signal used to diagnose and monitor cardiovascular disease, usually using 2- D ECG. Numerous studies have proven that ECG can be used to detect human emotions using 1-D ECG; however, ECG is typically captured as 2-D images rather than as 1-D data. There is still no consensus on the effect of the ECG input format on the accuracy of the emotion recognition system (ERS). The ERS using 2-D ECG is still inadequately studied. Therefore, this study compared ERS performance using 1-D and 2-D ECG data to investigate the effect of the ECG input format on the ERS.

**Methods: **This study employed the DREAMER dataset, which contains 23 ECG recordings obtained during audio-visual emotional elicitation. Numerical data was converted to ECG images for the comparison. Numerous approaches were used to obtain ECG features. The Augsburg BioSignal Toolbox (AUBT) and the Toolbox for Emotional feature extraction from Physiological signals (TEAP) extracted features from numerical data. Meanwhile, features were extracted from image data using Oriented FAST and rotated BRIEF (ORB), Scale Invariant Feature Transform (SIFT), KAZE, Accelerated-KAZE (AKAZE), Binary Robust Invariant Scalable Keypoints (BRISK), and Histogram of Oriented Gradients (HOG). Dimension reduction was accomplished using linear discriminant analysis (LDA), and valence and arousal were classified using the Support Vector Machine (SVM).

**Results: **The experimental results show 1-D ECG-based ERS achieved 65.06% of accuracy and 75.63% of F1 score for valence, and 57.83% of accuracy and 44.44% of F1-score for arousal. For 2-D ECG-based ERS, the highest accuracy and F1-score for valence were 62.35% and 49.57%; whereas, the arousal was 59.64% and 59.71%.

**Conclusions: **The results indicate that both inputs work comparably well in classifying emotions, which demonstrates the potential of 1-D and 2-D as input modalities for the ERS.

## Introduction

**Figure 1.**
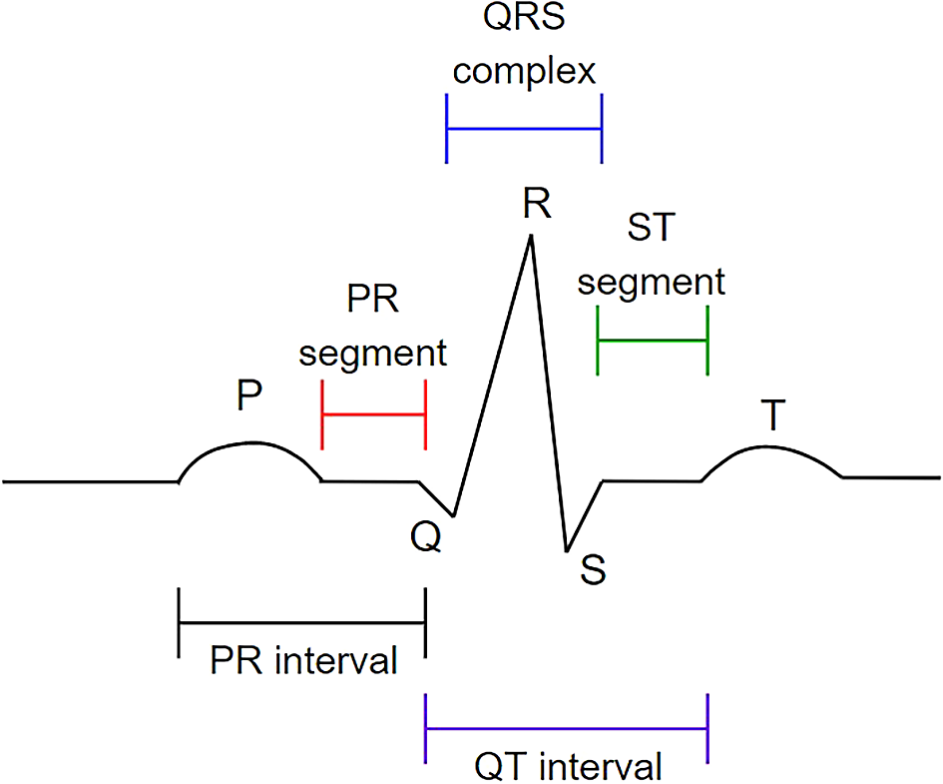
The P wave, QRS complex, and T wave in the standard electrocardiogram (ECG). This figure has been reproduced with permission from Ref. 7.

Medical professionals have been actively using electrocardiogram (ECG) wave images as a tool for monitoring ^1^ ^–^ ^3^ and diagnosing cardiovascular diseases, ^4^ ^–^ ^6^ such as heart attacks, dysrhythmia, and pericarditis, with some reported accuracy of more than 99% in the past decade. Fundamentally, ECG is used to measure electrical activity in the human heart by attaching electrodes to the human body. Due to the continual blood pumping action to the body, the electrical activity of the heart can be found in the sinoatrial node. The electrocardiogram signal is composed of three basic components: P, QRS, and T waves ( Figure 1). P waves are produced during atrium depolarization, QRS complexes are produced during ventricular depolarization, and T waves are produced during ventricle recovery.

Today’s ECG devices have advanced from large and immobile to compact, wearable, and portable. Additionally, the signal accuracy of portable devices is comparable to that of traditional medical devices and can be used for the same purposes as traditional devices, including the study of human emotions. Many studies have proven that ECG which is associated with autonomic nervous system’s (ANS) physiological responses can be used to identify human emotions. ^8^ ^–^ ^11^ Different emotions influence human heart activities differently; these influences may be hidden in the ECG wave and can be detected through closer monitoring of the main features of ECG, namely, heart rate (HR) and heart rate variability (HRV).

**Figure 2.**
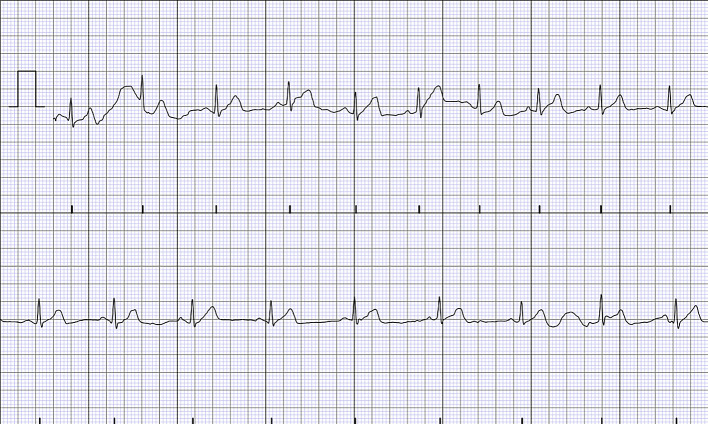
The example of 2-D ECG in a PDF file.

Previous research on human emotions has primarily relied on either direct analysis of 1-D data ^12^ ^–^ ^14^ or the conversion of 1-D data to a 2-D spectral image ^15^ prior to identifying the emotions. Despite this, majority of the portable devices record the ECG signal as images (2-D images) in a PDF file rather than as raw numerical data (1-D data). ^16^ ^–^ ^18^ The example of a PDF-based 2-D ECG is depicted in Figure 2. Due to this problem, researchers were required to convert the PDF file of the ECG into 1-D data before performing further emotion analysis, adding complexity to the pre-processing process. On this account, given the positive results obtained in monitoring and diagnosing cardiovascular-related diseases, the efficacy of 2-D ECG in emotion studies also warrants further investigation.

As far as our knowledge is concerned, despite numerous attempts to recognize emotions using ECG signals, the effects of employing various types of ECG inputs to recognise emotions in the emotion recognition system (ERS) have yet to be closely studied. In addition, there is no consensus on whether or not the type of ECG input format affects the emotion classification accuracy. Therefore, to address this gap, the contribution of this study is to compare emotion classification performance using 1-D and 2-D ECGs to investigate the effect of the ECG input format on the ERS.

This study analysed ECG data from the DREAMER dataset, a multimodal database. In DREAMER, ECG signals were recorded from 23 participants using 18 audio-visual stimuli for the elicitation of various emotions. The Augsburg BioSignal Toolbox (AUBT) ^19^ and the Toolbox for Emotional Feature Extraction from Physiological Signals (TEAP) ^20^ were used to help extract features from the 1-D ECG. Prior to emotion classification, the dimension of the extracted ECG features was reduced using linear discriminant analysis (LDA). On the other hand, the 2-D ECG was obtained by converting the 1-D ECG, and six different feature extractors were used to extract features from the 2-D ECG, namely Oriented FAST and Rotated BRIEF (ORB), Scale Invariant Feature Transform (SIFT), KAZE, Accelerated-KAZE (AKAZE), Binary Robust Invariant Scalable Keypoints (BRISK), and Histogram of Oriented Gradients (HOG). The Support Vector Machine (SVM) classifier is used, and the ERS results for both ECG inputs are compared to examine the effect of signal input on ERS performance. The finding indicates no substantial difference between the two ECG inputs since both produce a promising outcome within same range of accuracy for emotion recognition.

The next section discusses related works. The following section describes the dataset and the proposed methods in depth. The results are then provided. Finally, the study is concluded in the final section.

### Related works

Researchers in the emotion recognition field have been proposing multiple approaches using electrocardiogram signals. For instance, Minhad, Ali, and Reaz ^21^ used 1-D ECG to classify emotions of happiness and anger. They achieved 83.33% accuracy using the SVM classification method. Besides, Tivatansakul and Ohkura ^22^ used 1-D ECG from the AUBT dataset to detect emotions for the emotional healthcare system. K-Nearest Neighbour (KNN) successfully classified three emotions (joy, anger, and sadness) with an accuracy 85.75%, 82.75%, and 95.25%, respectively. The MPED database for ERS was proposed by Song *et al.* ^23^ using ECG numerical data to recognise discrete emotions (joy, humour, disgust, anger, fear, sadness, and neutrality). Attention Long Short-Term Memory (A-LSTM) was used as a feature extractor to extract the frequency and time-domain features from the physiological signal. The A-LSTM was used as a classifier along with SVM, KNN, and Long Short-Term Memory (LSTM). Averagely, A-LSTM achieved better results of 40% to 55% compared to those of other classifiers.

Katsigiannis and Ramzan ^13^ suggested that ERS should use low-cost and off-the-shelf devices to collect ECG signals based on numerical format. Their dataset was called DREAMER, and the classification using SVM with a radial basis function kernel successfully achieved 62.37% for valence and arousal. This dataset is adopted here. Additionally, numerous other researchers also used the ECG signals from the DREAMER dataset to perform emotion recognition. For instance, 1-D ECG data from the DREAMER dataset is utilized by Wenwen He et al. ^24^ that suggested an approach for emotion recognition using ECG contaminated by motion artefacts. The proposed approach improved classification accuracy by 5% to 15%. Additionally, Pritam and Ali ^25^ also employed 1-D ECG from the DREAMER dataset to develop the self-supervised deep multi-task learning framework ERS, which consists of two stages of learning: ECG representation learning and emotion classification learning. The accuracy gained in this study was greater than 70%. Hasnul et al. ^12^ also used the 1-D ECG by DREAMER dataset to compare the performance of two feature extractor toolboxes. They noted that the dataset’s size and the type of emotion classified might affect the suitability of the extracted features.

As mentioned before, the 2-D ECG was widely used for a variety of other purposes, including human authentication, ECG classification, and cardiovascular-related diseases. For example, Ref. 26 and Ref. 27 developed authentication systems based on printout-based 2-D ECG and 2-D ECG spectral images that achieved greater than 99% accuracy using CNN. Additionally, Klosowski et al. ^28^ reached the highest accuracy rate of 100% by classifying ECG signals into several categories, including normal ECG, brachy cardia, and premature ventricular contraction (PVC). Meanwhile, Ref. 4 and Ref. 29 employ 2-D ECG to detect and diagnose cardiovascular disease, specifically myocardial infarction (MI) and arrhythmia. Additionally, Mandal et al. ^5^ published a study comparing 1-D and 2-D ECGs for the diagnosis of Ventricular Arrhythmia. They concluded that both ECG inputs are effective at detecting the disease.

Despite rising popularity among medical practitioners in assessing patients’ cardiac disease, 2-D ECG remains inadequate compared to 1-D ECG usage as a type of input in emotion recognition studies. As a result, the number of studies employing 1-D ECG in ERS is higher than that utilizing 2-D ECG in ERS. However, rather than employing a printout-based 2-D ECG, emotion researchers classified human emotions using 2-D ECG spectral images. For example, Ref. 15 determines the R-peaks of the electrocardiogram prior to generating the R-R interval (RRI) spectrogram. Following that, CNN was used to classify the emotions, with an accuracy rate greater than 90%. Elalamy et al. ^30^ used ResNet-50 to extract features from a 2-D ECG spectrogram. Then, Logistic Regression (LR) was employed as a classifier and achieved an accuracy of 78.30% in classifying emotions.

Table 1 summarises these works, including the reference to the work, the dataset details (number of participants, number of stimuli), the signal used, the ECG input, the purpose of the work, the features extracted, the classifiers, and their accuracy—the accuracy denoted by an asterisk (*) refers to the accuracy of works that do not mainly focus on ERS.

**Table 1.** The summary of existing works using 1-D and 2-D ECG input.

Ref	Dataset	Signal used	ECG input	Purposes	Feature extracted	Classifier	Result (%)
21	Own dataset 69 subjects, 20 stimuli	ECG	1-D	ERS	Statistical features from the time and frequency domains	SVM, NB, KNN, Gaussian	SVM – 69.23 NB – 53.83 KNN – 61.83 Gaussian – 70.00
22	AUBT	ECG	1-D	ERS	Local pattern description using Local Binary Pattern (LBP) and Local Ternary Pattern (LTP)	KNN	LBP – 84.17 LTP – 87.92
23	(MPED) 23 subjects, 28 stimuli	ECG	1-D	ERS	Statistical features from the time and frequency domains	SVM, KNN, LSTM, A-LSTM	SVM – 42.66 KNN – 40.02 LSTM – 49.77 A-LSTM – 51.66
13	(DREAMER) 23 subject, 18 stimuli	ECG, EEG	1-D	ERS	Statistical features from the time and frequency domains	SVM, KNN, LDA	Valence – 62.37 Arousal – 62.37
24	DREAMER	ECG	1-D	ERS	Statistical features from the time, frequency, time-frequency domains, and nonlinear analysis-related	SVM	Valence – 86.09 Arousal – 87.80
25	DREAMER	ECG	1-D	ERS	Deep-learning	Convolutional Neural Network (CNN)	Valence – 74.90 Arousal – 77.10
12	DREAMER	ECG	1-D	ERS	Statistical features from the time and frequency domains	SVM	Valence – 65.80 Arousal – 65.40
26	(MWM-HIT) 100 subjects	ECG	2-D	Authentication System	PQRST peaks	CNN	99.99*
27	PhysioNet dataset (Fantasia and ECG-ID)	ECG	2-D spectral	Authentication system	Spectrogram	CNN	99.42*
28	Own dataset generated by FLUKE “ProSim 4 Vital Sign and ECG Simulator”	ECG	2-D spectral	ECG classification	Instantaneous frequency and spectral entropy	LSTM	100*
4	Zhejiang dataset	ECG	2-D	Myocardial infarction screening	Object detection	DenseNet, KNN, SVM	DenseNet – 94.73* KNN – 89.84* SVM – 92.19*
29	MIT-BIH arrhythmia dataset	ECG	2-D spectral	Arrhythmia classification	Local features from 2-D images using deep learning	CNN	99.11*
5	Physiobank dataset	ECG	1-D and 2-D	Ventricular Arrhythmia detection	ECG beat images	SVM, Probabilistic Neural Network (PNN), KNN, Random Forest (RF)	99.99* (both are useful)
15	Own dataset 11 subjects, 6 stimuli	ECG and EEG	2-D spectral	ERS	Statistical features from the time and frequency domains (R-R interval spectrogram)	CNN	ECG – 91.67 EEG – 90.00
30	AMIGOS, DEAP	ECG, PPG, EDA	2-D spectral	ERS	Features extracted from spectrogram by ResNet-50	Logistic Regression	AMIGOS – 78.30 DEAP – 69.45

Although considerable research has been conducted using ECG for ERS, the majority of research has focused on 1-D ECG analysis rather than 2-D ECG analysis, despite the fact that systems based on 2-D ECG have achieved excellent results in detecting cardiovascular-related diseases and human authentication. Additionally, no comparison of 1-D and 2-D ECG input was found in the emotion studies. As a result, it is unknown whether the ECG input format has an effect on the ERS’s emotional classification accuracy. The significance of this study is that it compares emotion classification performance between 1-D and 2-D ECGs to determine the effect of the ECG input format on the ERS.

## Methods

In this section, the details of the dataset are described, and the experimental setup for 1-D and 2-D ECGs is explained. The current study began in September 2020. MATLAB version 9.7 was utilized for data conversion and feature extraction, whereas Python version 3.8.5 was used for feature dimension reduction (for 1-D ECG) and classification. The analysis code used in this study is available from GitHub and archived with Zenodo. ^47^

### The dataset (DREAMER)

This study used ECG signals from Katsigiannis and Ramzan ^13^ called DREAMER. The DREAMER dataset is a freely accessible database of electroencephalogram (EEG) and electrocardiogram (ECG) signals used in emotion research. However, EEG signals were removed from this study because the primary focus is on ECG signals. The ECG was recorded using the SHIMMER ECG sensor at 256 Hz and stored in 1-D format. The DREAMER dataset contains 414 ECG recordings from 23 subjects who were exposed to 18 audio-visual stimuli designed to evoke emotion. Each participant assessed their emotions on a scale of 1 to 5 for arousal, valence, and dominance. However, because this study was primarily concerned with arousal and valence ratings, participants’ evaluations of dominance were discarded. The summary of the DREAMER dataset is tabulated in Table 2.

**Table 2.** The summary of the DREAMER dataset.

No of subject	23
**No of videos**	18 audio-visual stimuli
**Type of stimuli**	Audio-video
**Used Signal (Hz)**	ECG (256)
**Rating scales**	Valence, Arousal
**Rating values**	1–5

### Experimental setup

*1) 1-D ECG*

**Figure 3.**
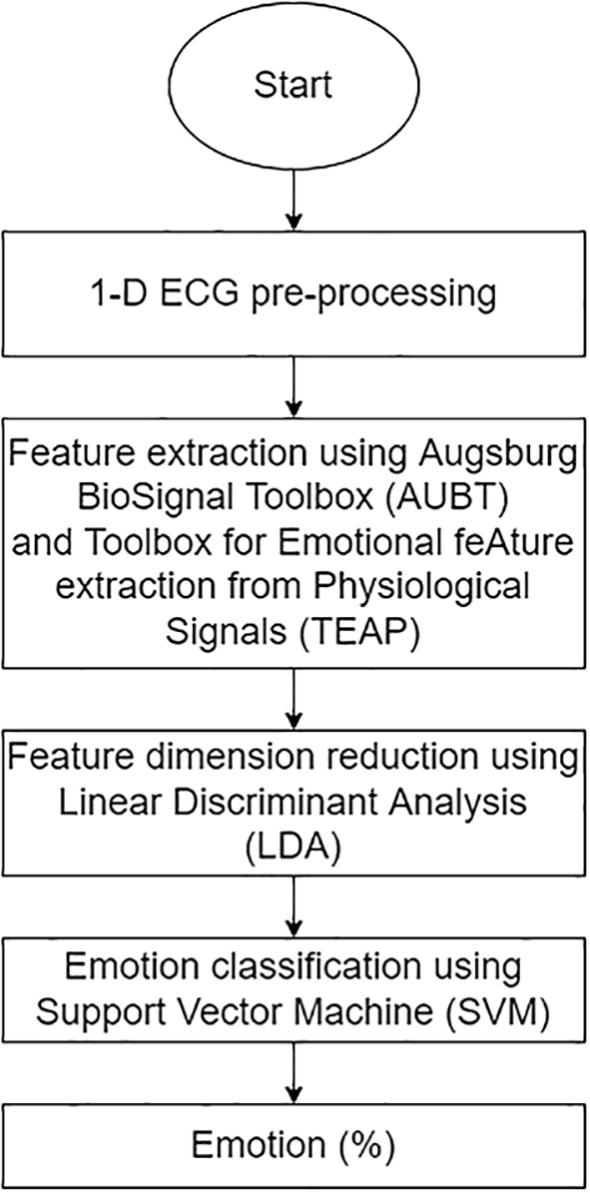
The overall structure of 1-D ECG-based ERS.

The proposed ERS for 1-D ECG consists of three stages: feature extraction, feature dimension reduction, and emotion classification. The structure of the proposed 1-D ECG-based ERS is illustrated in Figure 3.

Two open-source toolboxes, namely, Augsburg BioSignal Toolbox (AUBT) ^19^ and Toolbox for Emotional feature extraction from Physiological signals (TEAP), ^20^ were employed to facilitate feature extraction from the ECG signals. AUBT provides tools for the analysis of physiological signals such as the ECG, RESP, EMG, and GSR. These tools are available for Windows with MATLAB 7.1. On the other hand, TEAP is compatible with the MATLAB and Octave software packages operating on Windows and can analyse and compute features from physiological data such as EEG, GSR, PPG, and EMG.

The AUBT and TEAP feature extractors were included with the Low Pass Filter (LPF), a filter meant to reject all undesirable frequencies in a signal. The LPF was one of the most widely used filters before the computation of statistical features for physiological signals. ^31^ ^,^ ^32^ As a result, automated 1-D ECG pre-processing utilizing LPF was performed in this study to reduce muscle and respiratory noise in ECG signals.

AUBT extracted 81 features in the time and frequency domains from each 1-D ECG signal, including the mean, median, and standard deviation for each PQRST wave, HRV, frequency spectrum range, and amplitude signal. Sixteen (16) statistical features are extracted, including mean, IBI, HRV, and multiscale entropy in the time and frequency domains. Table 3 and Table 4 provide abbreviations and descriptions of AUBT and TEAP features, respectively.

**Table 3.** Features extracted from Augsburg Bio-signal Toolbox (AUBT).

Features	Description
P, Q, R, S, T	P-, Q-, R-, S-, T-peaks (ECG)
HRV	Heart rate variability
Ampl	Amplitude signal
Mean	Mean value
Median	Median value
Std	Standard deviation
Min	Minimum value
Max	Maximum value
SpecRange	Mean of the frequency spectrum in a given range

**Table 4.** Features extracted from Toolbox for Emotional feature extraction from Physiological signals (TEAP).

Features	Description
meanIBI	Mean inter-beat interval
HRV	Heart Rate Variability
MSE	Multiscale entropy at 5 levels
sp0001/0102/0203/0304	Spectral power 0-0.1 Hz, 0.1-0.2 Hz, 0.2-0.3 Hz, 0.3-0.4 Hz
energyRatio	Spectral energy ratio between f<0.08 Hz/f>0.15 Hz and f<5.0 Hz
tachogram_LF/MF/HF	Spectral power in tachogram (HRV) for low, medium, and high frequencies.
tachogram_energy_ratio	Energy ratio for tachogram spectral content (MF/(LF+HF))

Additionally, to prevent the “Curse of Dimensionality,” dimensionality reduction is employed to further reduce the number of high-dimensional features to low-dimensional features. The dimensions of the features were decreased using linear discriminant analysis (LDA), a well-known approach for reducing the dimensions of features. ^33^ LDA is a supervised algorithm that can reduce dimensionality while retaining as much class-discrimination information as possible. Following that, the low-dimensional features were fed into a Support Vector Machine (SVM) classifier for emotion classification. The following section will outline the classifying process.

*2) 2-D ECG*

**Figure 4.**
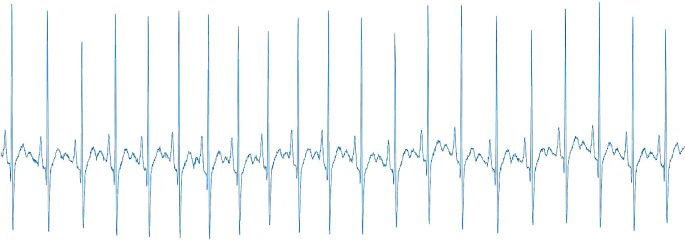
The 2-D ECG converted from 1-D ECG.

The duration of the ECG recording varies according to the duration of the video (average = 199 seconds). As Katsigiannis and Ramzan proposed, this study analysed the final 60 seconds of each recording to allow time for a dominant emotion to emerge. ^13^ Following that, 1-D ECG was pre-processed using a simple MATLAB function by Ref. 34 to eliminate baseline wander caused by breathing, electrically charged electrodes, or muscle noise. The signal was then divided into four segments corresponding to 15 seconds each. Then, using MATLAB version 9.7, the 1-D ECG was transformed into a 2-D ECG ( Figure 4). The image has a width of 1920 pixels and a height of 620 pixels.

Due to the fact that the 2-D ECG was converted to a rectangle shape, it is not easy to resize the photos to the standard input image sizes of 224×224 and 299×299. As a result, the converted 2-D ECG was resized to 60% of its original size using Python version 3.8.5. This scale percentage was chosen after considering the quality of the image, the type of feature extractor used, and the computational cost the system can afford. The coloured images were converted into greyscale images. Then, binarization of the image using an Otsu’s automatic image thresholding method ^35^ was done. This method ascertains the optimal threshold values from pixel values of 0 to 255 by calculating and evaluating their within-class variance. ^36^

The area of interest for 2-D ECG is laying on the PQRST waves, making the peaks detector the best approach to be employed. Therefore, six different feature extractors that could extract peaks, edges, or corners were applied to extract features from 2-D ECGs using Python version 3.8.5:

1. ORB ^37^: ORB features are invariant to rotation and noise because they are a combination of Features from Accelerated Segment Test (FAST) detection and Binary Robust Independent Elementary Features (BRIEF) description methods.
2. SIFT ^38^: SIFT identifies feature points by searching for local maxima on the images using Difference-of-Gaussians (DoG) operators. The description approach generates a 16x16 neighbourhood around each identified feature and sub-blocks the region. SIFT is also rotation and scale invariant.
3. KAZE ^39^: KAZE is based on the scale of the normalised determinant of the Hessian Matrix, with the maxima of detector responses being captured as feature points using a moving window. Additionally, KAZE makes use of non-linear space via non-linear diffusion filtering to reduce noise while keeping the borders of regions in images.
4. AKAZE ^40^: AKAZE is a more sophisticated version of KAZE that is based on the Hessian Matrix determinant. Scharr filters were employed to enhance the quality of the invariance rotation, rendering AKAZE features rotation- and scale-invariant.
5. BRISK ^41^: While searching for maxima in the scale-space pyramid, BRISK detects corners using the AGAST algorithm and filters them using the FAST Corner Score. Additionally, the BRISK description is based on the recognised characteristic direction of each feature, which is necessary for rotation invariance.
6. HOG ^42^: HOG is a feature descriptor that is used to compute the gradient value for each pixel. The image shape denoted the edge or gradient structure derived using a high-quality local gradient intensity distribution.

**Figure 5.**
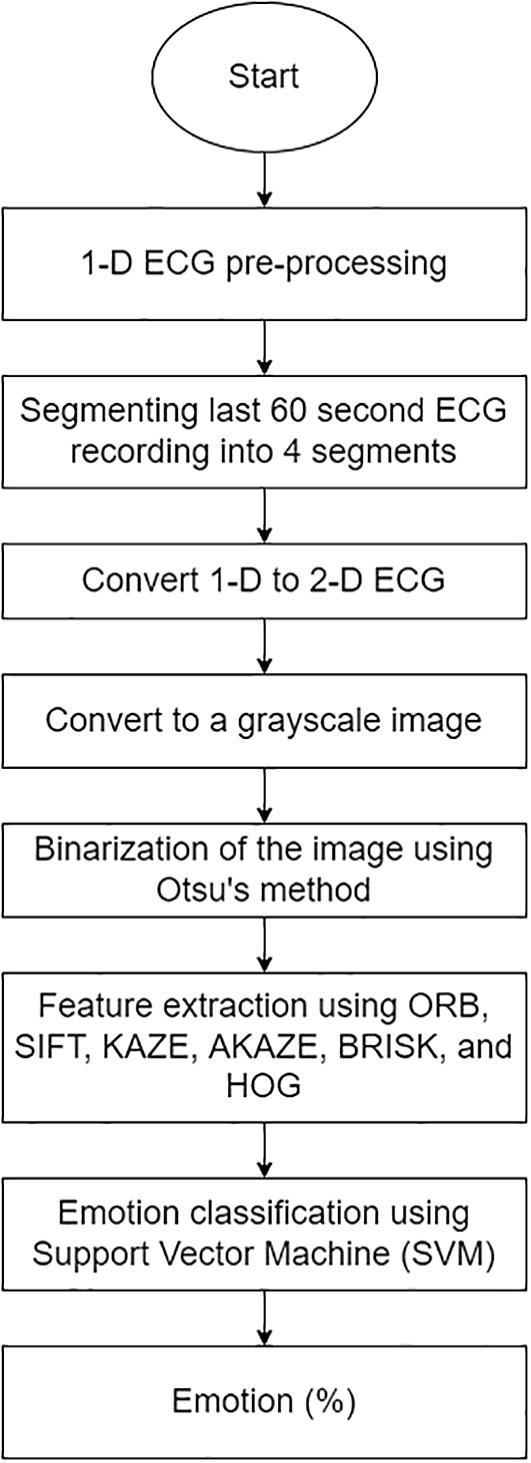
The overall structure of 1-D ECG-based ERS.

All of the extractors successfully extracted the ECG features, including the peaks, edges, and corners from the PQRST waves. The extracted features were then given to the classifier (SVM) to classify the emotions. Figure 5 illustrates the structure of the proposed 2-D ECG-based.

### Support vector machine

Emotion classification was performed using SVM. The SVM works by separating the class data points and drawing a boundary called the hyperplane between them. Each hyperplane has what are known as “decision boundaries” to determine which side of each class resides. As reported in previous studies, SVM has a low computational cost and shows excellent performance in classifying emotions, as reported in previous studies. ^13^ ^,^ ^21^ ^,^ ^24^ ^,^ ^43^ ^,^ ^44^

### Experimental setting

The scale of self-assessed emotions, which ranges from 1 (lowest) to 5 (highest), was classified using a five-point scale with middle-point thresholds (an average rate of 3.8). As a result, scales four and five were assigned to the high class, while the remaining scales were assigned to the low class. This results in an imbalanced distribution of DREAMER classes: valence has 39% of high valence and 61% of low valence; arousal has 44% of low arousal and 56% of high arousal.

The hyperparameters for SVM were tuned using an exhaustive parameter search tool, GridSearchCV, from Scikit-learn that automates the tuning procedure. ^45^ This study tuned only the parameters with a high and relative tuning risk and left the remainder at their default values because they are the least sensitive to the hyperparameter tuning process, as suggested by Weerts, Mueller, and Vanschoren. ^46^

The dataset was split into a reasonable proportion of training and testing sets to evaluate the model’s performance on new unseen data. This study used a stratified train-test split of 80:20 for the training and testing sets. This strategy guarantees the dataset’s exact proportions of samples in each class are preserved.

Additionally, as we had a small dataset size, this study applied KFold Cross-Validation, with the number of folds set to 10, the most commonly used number in prior research to improve ERS performance. The experimental setting is tabulated in Table 5.

**Table 5.** The experimental setting values.

Setting		Value
SVM hyperparameter	Kernel	{linear, rbf}
	C	[0.1,1,10]
	Gamma	[0.1,1,10]
Train-test split		Stratified 70:30
Kfold cross-validation		10

## Results

The testing performance of the ERS in classifying emotions using two different types of ECG data, 1-D and 2-D, is summarised in Table 6. The result denoted by an asterisk (*) corresponds to the original DREAMER publication 13, whereas the best accuracy and F1 score for classifying valence and arousal were bolded and shaded.

**Table 6.** Testing emotion classification accuracy and F1-score for 1-D and 2-D electrocardiogram (ECG).

Type of ECG input	Feature extractor	Valence		Arousal	
		Accuracy	F1-Score	Accuracy	F1-Score
1-D	*AUBT and BioSig	62.37	53.05	62.37	57.98
	AUBT	63.86	72.73	*57.83*	*44.44*
	TEAP	*65.06*	*75.63*	54.22	42.42
2-D	ORB	61.75	47.76	56.33	40.59
	KAZE	*62.35*	*49.57*	56.33	40.59
	SIFT	61.14	46.4	56.33	40.59
	AKAZE	61.75	47.76	*59.64*	*59.71*
	BRISK	61.75	47.76	56.02	40.23
	HOG	61.14	46.4	56.33	40.59

For 1-D input, the features extracted using the TEAP feature extractor obtain the best valence performance with an accuracy of 65.06% and an F1 score of 75.63%. The best arousal performance is obtained using features extracted by the AUBT feature extractor, which has a 57.83% and a 44.44% F1 score.

On the other hand, the KAZE feature extractor achieves the best valence performance with 2-D input, achieving 62.35% accuracy and a 49.57% F1 score. Simultaneously, with 59.64% accuracy and a 59.71% F1 score, the AKAZE feature extractor achieves the best performance in arousal emotion.

For comparison purposes, the computation time for both ECG inputs was recorded and reported in Table 7. The average time required to compute 1-D is 1.58 ± 0.07 seconds. In comparison, the average computation time for 2-D is 3377.425 ± 3138.875 seconds. Therefore, according to the observation, 2-D took the longest computation time, whereas 1-D obtained the shortest.

**Table 7.** Computation Time for Each Feature Extractor using Support Vector Machine (SVM).

Type of ECG input	Feature extractor	Computational time (sec)	
		Valence	Arousal
**1-D**	AUBT	1.65	1.63
	TEAP	1.51	1.55
**2-D**	ORB	1473.07	1461.77
	KAZE	4486.27	6034.26
	SIFT	239.31	238.55
	AKAZE	2950.23	3308.23
	BRISK	4926.46	3610.64
	HOG	6516.30	6431.28

## Discussion & conclusions

The results indicate that both inputs work comparably well in classifying emotions. This finding is demonstrated by the fact that the best valence performance was obtained using a 1-D ECG, and the best arousal performance was acquired using a 2-D ECG. Additionally, ERS with 1-D ECG was combined with dimensionality reduction, called LDA. The presence of LDA improved the ERS performance in valence emotion but not in arousal. In terms of computational cost, 1-D ECG is better to 2-D ECG since it requires less computation time.

However, it is worth mentioning that the results obtained using 2-D ECG demonstrated potential for use as an input modality for the ERS. Additionally, 2-D ECGs are appealing because the format enables the use of a variety of image-based methods such as image augmentation to increase the data size, convolution neural networks (CNN), and the application of transfer learning from models trained using large data. To summarise, the ERS performance of the two ECG inputs is comparable since both yield a promising outcome for emotion recognition.

## Data availability

### Source data

The DREAMER dataset was first presented here: https://doi.org/10.1109/JBHI.2017.2688239 and can be found on Zenodo. Access is restricted and users are required to apply. The decision whether to grant/deny access is solely under the responsibility of the record owner.

### Extended data

Analysis code available from: https://github.com/nr-isml/ECG-Numerical-Vs.-Image-Data-for-Emotion-Recognition-System

Archived analysis code as at time of publication: https://doi.org/10.5281/zenodo.5542739. ^47^

License: Data are available under the terms of the Creative Commons Zero “No rights reserved” data waiver (CC0 1.0 Public domain dedication).
