# Supplementary material for: Evaluation of electrocardiogram: numerical vs. image data for emotion recognition system
Source: F1000Res. 2022 May 30;10:1114. Originally published 2021 Nov 4. [Version 2] doi: 10.12688/f1000research.73255.2 (PMC9171287; doi:10.12688/f1000research.73255.2)
Supplement: Peer review history

# Peer review: Reviewer response for version 2

**DOI:** 10.5256/f1000research.134293.r139365

**Published:** 2022-06-06

**Authors:** Marios Fanourakis

**Affiliations:** 1 University of Geneva, Geneva, Switzerland

After evaluating the author's revisions and replies to my previous comments I would like to upgrade the status to Approved.

 The authors have carefully addressed the issues previously identified with only some minor clarifications needed.

 What method was used to resize/rescale the 2D ECG images (which python function)? I see in the github code that the following method was used "cv2.resize(img, dim, interpolation = cv2.INTER_AREA)", please explain what the interpolation method cv2.INTER_AREA is.

 As mentioned the 2D image is 1920x620 pixels, a 60% rescaling doesn't result in either 224x224 nor 299x299. Some information is still missing here. Furthermore, why is 224x224 and 299x299 a "standard size"? Is it that these sizes are common in other ML models? Other reasons?

 When splitting the data between the training and test set, was an individual participant's data also split between the train and test sets? For example, suppose there is a participant "A", would one find some data of participant "A" in the training set and some other data of the same participant "A" in the test set, or was it split such that if there was data of participant "A" in the training set there was no data from participant "A" in the test set and vice versa? This could help to further interpret the results.

 From a quick glance at the github code, it seems that participants' data could be present in both training and test sets. This is not inherently bad and I don't believe it invalidates the results and conclusions presented, especially since the work's main contribution is to compare the performance of 1D ECG signals to 2D ECG images as inputs to ML models. Nonetheless, it is important to mention.

 A short definition of "stratified train-test split" could be helpful for readers.

Is the work clearly and accurately presented and does it cite the current literature?

No

If applicable, is the statistical analysis and its interpretation appropriate?

Not applicable

Are all the source data underlying the results available to ensure full reproducibility?

Yes

Is the study design appropriate and is the work technically sound?

Partly

Are the conclusions drawn adequately supported by the results?

Partly

Are sufficient details of methods and analysis provided to allow replication by others?

No

Reviewer Expertise:

Affective computing, emotion recognition

I confirm that I have read this submission and believe that I have an appropriate level of expertise to confirm that it is of an acceptable scientific standard.

# Peer review: Reviewer response for version 2

**DOI:** 10.5256/f1000research.134293.r139366

**Published:** 2022-05-31

**Authors:** Md. Asadur Rahman

**Affiliations:** 1 Department of Biomedical Engineering, Military Institute of Science and Technology (MIST), Dhaka, Bangladesh

The revised version can be approved. I am satisfied so far.

Is the work clearly and accurately presented and does it cite the current literature?

Yes

If applicable, is the statistical analysis and its interpretation appropriate?

Yes

Are all the source data underlying the results available to ensure full reproducibility?

Yes

Is the study design appropriate and is the work technically sound?

Yes

Are the conclusions drawn adequately supported by the results?

Partly

Are sufficient details of methods and analysis provided to allow replication by others?

Yes

Reviewer Expertise:

NA

I confirm that I have read this submission and believe that I have an appropriate level of expertise to confirm that it is of an acceptable scientific standard.

# Peer review: Reviewer response for version 1

**DOI:** 10.5256/f1000research.76896.r121059

**Published:** 2022-03-16

**Authors:** Md. Asadur Rahman

**Affiliations:** 1 Department of Biomedical Engineering, Military Institute of Science and Technology (MIST), Dhaka, Bangladesh

The paper titled “Evaluation of electrocardiogram: numerical vs. image data for emotion recognition system” looks interesting. Although it is a short paper, I found it interesting and am positive about this work, but it should go through some modification:

- Figure 2 should be redrawn, only providing the reference is not enough to present a figure or image in the article.
- The ECG signals used in this work contain some baseline wandering. It should be removed before further analysis. A simple technique with Matlab code is described in Rahman *et al*  (2019) ^1^ to remove baseline wander. I am expecting a result comparing the emotion recognition rate before and after the baseline wander removal from the ECG signal.
- In addition to that, is it possible to use LSTM to the ECG time series to find the accuracy of the emotion recognition from ECG signals?
- Please provide detailed information about transforming the signal to image conversion.
- The reference-related comparison and discussion should be completed in the result and discussions. If possible, avoid referencing in the Conclusion section.

Is the work clearly and accurately presented and does it cite the current literature?

Yes

If applicable, is the statistical analysis and its interpretation appropriate?

Yes

Are all the source data underlying the results available to ensure full reproducibility?

Yes

Is the study design appropriate and is the work technically sound?

Yes

Are the conclusions drawn adequately supported by the results?

Partly

Are sufficient details of methods and analysis provided to allow replication by others?

Yes

Reviewer Expertise:

Biomedical Signal Processing

I confirm that I have read this submission and believe that I have an appropriate level of expertise to confirm that it is of an acceptable scientific standard, however I have significant reservations, as outlined above.

## References

[1] : A statistical designing approach to MATLAB based functions for the ECG signal preprocessing. *Iran Journal of Computer Science* .2019;2(3) : 10.1007/s42044-019-00035-0 167-178 10.1007/s42044-019-00035-0

# Response

**Published:** 2022-05-20

**Authors:** Sharifah Noor Masidayu Sayed Ismail

**The paper titled “Evaluation of electrocardiogram: numerical vs. image data for emotion recognition system” looks interesting. Although it is a short paper, I found it interesting and am positive about this work, but it should go through some modification:**

 We appreciate your feedback and suggestions. We have revised the manuscript as needed.

**Figure 2 should be redrawn, only providing the reference is not enough to present a figure or image in the article.**

 Thank you for your suggestions. As per your recommendations, we have redrawn the figure and an additional note has been added in the legend. As several modifications were made to the manuscript, Figure 2 was originally changed to Figure 1. This figure can be found on page 4.

**The ECG signals used in this work contain some baseline wandering. It should be removed before further analysis. A simple technique with MATLAB code is described in Rahman et al (2019)1 to remove baseline wander. I am expecting a result comparing the emotion recognition before and after the baseline wander removal from the ECG signal.**

 Thank you for your suggestion. We did use the method suggested as cited accordingly. However, your expectation of having a result comparing the emotion recognition before and after the baseline wander removal from the ECG signal is inappropriate for our work because the focus of this paper is to compare emotion classification performance using 1-D and 2-D ECGs to investigate the effect of the ECG input format on the ERS.

**In addition to that, is it possible to use LSTM to the ECG time series to find the accuracy of the emotion recognition from ECG signals?**

 As far as we know, it is possible to use LSTM on the ECG time series to find the accuracy of emotion recognition from ECG signals. The work by Song et al. used LSTM for the same purpose as your concern. Below is the paper, which you can take a look at. We hope it answers your question.

 T. Song, W. Zheng, C. Lu, Y. Zong, X. Zhang, and Z. Cui, “MPED: A multi-modal physiological emotion database for discrete emotion recognition,” IEEE Access, vol. 7, no. October, pp. 12177–12191, 2019.

**Please provide detailed information about transforming the signal to image conversion.**

 Thank you for pointing this out. We have revised the 2-D ECG subsection, where the pre-processing process until the transformation from 1-D to 2-D is explained in detail. This subsection can be found on pages 11 and 12, which can be read as follows:

 “The duration of the ECG recording varies according to the duration of the video (average = 199 seconds). As Katsigiannis and Ramzan proposed, this study analysed the final 60 seconds of each recording to allow time for a dominant emotion to emerge ^13^. Following that, 1-D ECG was pre-processed using a simple MATLAB function by ^34^ to eliminate baseline wander caused by breathing, electrically charged electrodes, or muscle noise. The signal was then divided into four segments corresponding to 15 seconds each. Then, using MATLAB version 9.7, the 1-D ECG was transformed into a 2-D ECG (Figure 4). The image has a width of 1920 pixels and a height of 620 pixels.

 Due to the fact that the 2-D ECG was converted to a rectangle shape, it is not easy to resize the photos to the standard input image sizes of 224×224 and 299×299. As a result, the converted 2-D ECG was resized to 60% of its original size using Python version 3.8.5. This scale percentage was chosen after considering the quality of the image, the type of feature extractor used, and the computational cost the system can afford. The coloured images were converted into greyscale images. Then, binarization of the image using an Otsu's automatic image thresholding method ^35^ was done. This method ascertains the optimal threshold values from pixel values of 0 to 255 by calculating and evaluating their within-class variance ^36^”

**The reference-related comparison and discussion should be completed in the result and discussions. If possible, avoid referencing in the Conclusion section.**

 Thank you for bringing this to our attention. We found your comments extremely helpful and have revised them accordingly. The revised text can be read as follows:

 “The results indicate that both inputs work comparably well in classifying emotions. This finding is demonstrated by the fact that the best valence performance was obtained using a 1-D ECG, and the best arousal performance was acquired using a 2-D ECG. Additionally, ERS with 1-D ECG was combined with dimensionality reduction, called LDA. The presence of LDA improved the ERS performance in valence emotion but not in arousal. In terms of computational cost, 1-D ECG is better to 2-D ECG since it requires less computation time.

 However, it is worth mentioning that the results obtained using 2-D ECG demonstrated potential for use as an input modality for the ERS. Additionally, 2-D ECGs are appealing because the format enables the use of a variety of image-based methods such as image augmentation to increase the data size, convolution neural networks (CNN), and the application of transfer learning from models trained using large data. To summarise, the ERS performance of the two ECG inputs is comparable since both yield a promising outcome for emotion recognition.”

# Peer review: Reviewer response for version 1

**DOI:** 10.5256/f1000research.76896.r126091

**Published:** 2022-03-16

**Authors:** Marios Fanourakis

**Affiliations:** 1 University of Geneva, Geneva, Switzerland

The authors use the DREAMER dataset to compare the emotion recognition performance of features extracted from the time-series ECG signal versus features extracted from images of the ECG wave signal. An SVM model is used as the classifier.

 Overall, the structure of the report is not coherent, the related work is incomplete, and the motivation for this work is not convincing. Furthermore, many important details are missing about the dataset and the methods used which makes it difficult to trust the results and the comparisons they make with other works.

 Detailed comments:

 Structure: several improvements to be made, information seems to be scattered throughout the article. For example, information about the dataset is present in both the introduction and methods sections. It is best to keep this information in the same section. Same for the related works.

 Authors should give a brief explanation on what are ECG wave images in the introduction, otherwise readers in the emotion recognition field might confuse them with spectrograms which are more widely used in the field. It may also be better to change the term "numerical data" to "time-series data" or "1D data" (2D being an image).

 Typo on page 3: EGC instead of ECG.

 From the references pertaining to the use of ECG images (1-6, 23,24) only half actually use ECG wave images (2,4,23,24). The rest either use time-series or convert the time-series to spectrograms. The ones that do use ECG images, mainly analyze individual beats and not the entire ECG wave in order to detect medical heart issues. For the emotion recognition use-case it is necessary to analyze significantly larger regions of the signal than individual beats.

 In emotion recognition, it is common to transform the ECG signal into a spectrogram image. The authors do not cite any work mentioning this method.

 Authors do not include any references to other works that use the DREAMER dataset ECG signals for emotion recognition, here are some:

 Wenwen He et al. 2021 Emotion Recognition from ECG Signals Contaminated by Motion Artifacts ^1^

 Pritam Sarkar et al. 2020 Self-supervised ECG Representation Learning for Emotion Recognition ^2^

 I also came across another publication of some of the co-authors which would be advantageous to reference:

 Muhammad Anas Hasnul et al. 2021 Evaluation of TEAP and AuBT as ECG’s Feature Extraction Toolbox for Emotion Recognition System ^3^

 In the Emotion model paragraph (page 3): it is still unclear which model of emotions was used, Ekman or Russell? Authors say "the latter" (referring to Russel) but then mention binary classification which may be confusing since the arousal/valence space is continuous making it a regression problem or at least multiclass and not binary. Authors should re-word this part to make it clear.

 Referring to wearable ECG devices, the authors state: "However, most of these devices store the ECG as images instead of raw numerical data". No references or other market analysis is provided to show that this is the case. The use of ECG wave images for emotion recognition is not properly motivated. I fail to see any advantage of using ECG wave images over the time-series data unless ECG time-series data is not available.

 How was the data annotated in the DREAMER dataset? Were they continuous annotations or a single annotation per video clip? This is very relevant to include in the article. A few more words about the dataset are needed like a short description of the experimental protocol.

 Arousal/valence rating values are ranging from 1 to 5 in the DREAMER dataset. The authors never explain how they split them into two classes (high/low) for the binary classification.

 Authors do not include sufficient information on how the time-series ECG data was converted to an image (resolution, compression, windowing), or how the data was treated in general. Did the authors use the entire ECG signal for each video? Was there any windowing? Since the authors did not properly summarize the dataset (how were the videos annotated?) it is difficult to grasp or guess on how the data was processed.

 None of the cited literature used any of the image feature extraction methods that the authors used, and the authors did not discuss their reasoning for why they selected those image feature extraction methods and not the ones established in the ECG image analysis literature that they cited. Some more illustrations of these features may be useful besides the one in Figure 5 in order to convince the readers.

 Support vector machine: It is not clear what preprocessing steps were applied to the data. For example in the DREAMER dataset baseline they only use the last 60s of data for each film clip.

 Data was divided 80:20 and 10-fold cross-validation was used. The authors do not specify exactly how the data was split (see DREAMER dataset paper section V as an example). From reading this part, I can only assume that all data from all participants was used for each fold (general model), something that is diverging from how the data was split in the DREAMER dataset paper (they made models for each individual participant). Therefore any comparisons to the results of the DREAMER baseline are invalid.

 Results: If the classes are unbalanced (as the DREAMER dataset paper indicates) accuracy is not valid on it's own, include f1 score, and/or Cohen's Kappa.

 Discussion: "Ref. 11" has nothing to do with the statement in that paragraph. LDA was actually applied in the DREAMER dataset paper and they reported that there were no significant differences in performance.

Is the work clearly and accurately presented and does it cite the current literature?

No

If applicable, is the statistical analysis and its interpretation appropriate?

Not applicable

Are all the source data underlying the results available to ensure full reproducibility?

Yes

Is the study design appropriate and is the work technically sound?

Partly

Are the conclusions drawn adequately supported by the results?

Partly

Are sufficient details of methods and analysis provided to allow replication by others?

No

Reviewer Expertise:

Affective computing, emotion recognition

I confirm that I have read this submission and believe that I have an appropriate level of expertise to state that I do not consider it to be of an acceptable scientific standard, for reasons outlined above.

## References

[1] : Emotion Recognition from ECG Signals Contaminated by Motion Artifacts,. *International Conference on Intelligent Technology and Embedded Systems (ICITES), 2021* .2021; 10.1109/ICITES53477.2021.9637072 125-130 10.1109/ICITES53477.2021.9637072

[2] : Self-supervised ECG Representation Learning for Emotion Recognition. *IEEE Transactions on Affective Computing* .2021; 10.1109/TAFFC.2020.3014842 10.1109/TAFFC.2020.3014842

[3] : Evaluation of TEAP and AuBT as ECG's Feature Extraction Toolbox for Emotion Recognition System.2021 IEEE 9th Conference on Systems, Process and Control (ICSPC 2021), 2021; 10.1109/ICSPC53359.2021.9689133 52-57 10.1109/ICSPC53359.2021.9689133

# Response

**Published:** 2022-05-20

**Authors:** Sharifah Noor Masidayu Sayed Ismail

**The authors use the DREAMER dataset to compare the emotion recognition performance of features extracted from the time-series ECG signal versus features extracted from images of the ECG wave signal. An SVM model is used as the classifier.**

**Overall, the structure of the report is not coherent, the related work is incomplete, and the motivation for this work is not convincing. Furthermore, many important details are missing about the dataset and the methods used which makes it difficult to trust the results and the comparisons they make with other works** **.**

 Thanks for taking the time to review our manuscript. We have revised the manuscript based on your comments and suggestions accordingly.

**Structure: several improvements to be made, information seems to be scattered throughout the article. For example, information about the dataset is present in both the introduction and methods sections. It is best to keep this information in the same section. Same for the related works** **.**

 Thanks for pointing this out. We agree with your suggestion and have attempted to address the issues. Therefore, we have revised each section as per suggestions by revising the information written in the manuscript accordingly. The revised part can be found on pages 3 until 13.

**Authors should give a brief explanation on what are ECG wave images in the introduction, otherwise readers in the emotion recognition field might confuse them with spectrograms which are more widely used in the field. It may also be better to change the term "numerical data" to "time-series data" or "1D data" (2D being an image)** **.**

 Thank you for drawing our attention to this. As you mentioned, we have added an explanation of what ECG wave images are in the Introduction section that reads as follows:

 “Fundamentally, ECG is used to measure electrical activity in the human heart by attaching electrodes to the human body. Due to the continual blood pumping action to the body, the electrical activity of the heart can be found in the sinoatrial node. The electrocardiogram signal is composed of three basic components: P, QRS, and T waves (Figure 1). P waves are produced during atrium depolarization, QRS complexes are produced during ventricular depolarization, and T waves are produced during ventricle recovery.

 Despite this, majority of the portable devices record the ECG signal as images (2-D images) in a PDF file rather than as raw numerical data (1-D data) ^16 – 18^. The example of a PDF-based 2-D ECG is depicted in Figure 2. Due to this problem, researchers were required to convert the PDF file of the ECG into 1-D data before performing further emotion analysis, adding complexity to the pre-processing process. On this account, given the positive results obtained in monitoring and diagnosing cardiovascular-related diseases, the efficacy of 2-D ECG in emotion studies also warrants further investigation.”

 Additionally, we also added Figure 2 that shows the snippet of the 2-D ECG from the PDF file. This figure can be found on page 4.

 Furthermore, as per suggestion, we have changed the terms "numerical data" to "1D ECG" and "wave images" to "2-D ECG".

**Typo on page 3: EGC instead of ECG** **.**

 Thank you so much for catching these glaring and confusing errors, which we have now corrected.

**From the references pertaining to the use of ECG images (1-6, 23,24) only half actually use ECG wave images (2,4,23,24). The rest either use time-series or convert the time-series to spectrograms. The ones that do use ECG images, mainly analyze individual beats and not the entire ECG wave in order to detect medical heart issues. For the emotion recognition use-case it is necessary to analyze significantly larger regions of the signal than individual beats** **.**

 Based on your comment, we have revised our Related Works section and corrected them accordingly. Additionally, we added the summary of existing works that use 1-D and 2-D ECG input with their purposes, tabulated in Table 1. The revision of these issues can be found on page 4 until 8.

**In emotion recognition, it is common to transform the ECG signal into a spectrogram image. The authors do not cite any work mentioning this method** **.**

 Thanks for pointing this out. We agree with your comments. Therefore, we have revised our Related Works section and provided improvements through the fourth paragraph on page 6:

 .

 “Despite rising popularity among medical practitioners in assessing patients' cardiac disease, 2-D ECG remains inadequate compared to 1-D ECG usage as a type of input in emotion recognition studies. As a result, the number of studies employing 1-D ECG in ERS is higher than that utilizing 2-D ECG in ERS. However, rather than employing a printout-based 2-D ECG, emotion researchers classified human emotions using 2-D ECG spectral images. For example, ^15^ determines the R-peaks of the electrocardiogram prior to generating the R-R interval (RRI) spectrogram. Following that, CNN was used to classify the emotions, with an accuracy rate greater than 90%. Elalamy et al. ^30^ used ResNet-50 to extract features from a 2-D ECG spectrogram. Then, Logistic Regression (LR) was employed as a classifier and achieved an accuracy of 78.30% in classifying emotions.”

 Additionally, in Table 1, the ECG input was listed as either 1-D or 2-D ECGs, where 2-D was further categorised into standard 2-D ECG or spectral 2-D ECG.

**Authors do not include any references to other works that use the DREAMER dataset ECG signals for emotion recognition, here are some:**

****

**Wenwen He et al. 2021 Emotion Recognition from ECG Signals Contaminated by Motion Artifacts**

**Pritam Sarkar et al. 2020 Self-supervised ECG Representation Learning for Emotion Recognition**

****

**I also came across another publication of some of the co-authors which would be advantageous to reference:**

****

**Muhammad Anas Hasnul et al. 2021 Evaluation of TEAP and AuBT as ECG’s Feature Extraction Toolbox for Emotion Recognition System** **.**

 Thank you for the paper suggested. We have added the reference according to your recommendation, which you can find on page 6 as follows:

 “Additionally, numerous other researchers also used the ECG signals from the DREAMER dataset to perform emotion recognition. For instance, 1-D ECG data from the DREAMER dataset is utilized by Wenwen He et al. ^24^ that suggested an approach for emotion recognition using ECG contaminated by motion artefacts. The proposed approach improved classification accuracy by 5% to 15%. Additionally, Pritam and Ali ^25^ also employed 1-D ECG from the DREAMER dataset to develop the self-supervised deep multi-task learning framework ERS, which consists of two stages of learning: ECG representation learning and emotion classification learning. The accuracy gained in this study was greater than 70%. Hasnul et al. ^12^ also used the 1-D ECG by DREAMER dataset to compare the performance of two feature extractor toolboxes. They noted that the dataset's size and the type of emotion classified might affect the suitability of the extracted features.”

**In the Emotion model paragraph (page 3): it is still unclear which model of emotions was used, Ekman or Russell? Authors say "the latter" (referring to Russel) but then mention binary classification which may be confusing since the arousal/valence space is continuous making it a regression problem or at least multiclass and not binary. Authors should re-word this part to make it clear** **.**

 Thank you for pointing this out. The reviewer is correct; the original phrase is confusing. Therefore, we have removed this part to avoid any further confusion. Furthermore, this concern has been changed and revised to make it more straightforward and understandable. The revised text was located in the experimental setting subsection under the Method section on page 13:

 “The scale of self-assessed emotions, which ranges from 1 (lowest) to 5 (highest), was classified using a five-point scale with middle-point thresholds (an average rate of 3.8). As a result, scales four and five were assigned to the high class, while the remaining scales were assigned to the low class.”

**Referring to wearable ECG devices, the authors state: "However, most of these devices store the ECG as images instead of raw numerical data". No references or other market analysis is provided to show that this is the case. The use of ECG wave images for emotion recognition is not properly motivated. I fail to see any advantage of using ECG wave images over the time-series data unless ECG time-series data is not available.**

 Thank you for drawing our attention to this, and we agree with the comments. Therefore, we have cited the necessary references and revised the statement as per the suggestion. The revised text can be found on page 4 as follows:

 “Previous research on human emotions has primarily relied on either direct analysis of 1-D data ^12 – 14^ or the conversion of 1-D data to a 2-D spectral image ^15^ prior to identifying the emotions. Despite this, majority of the portable devices record the ECG signal as images (2-D images) in a PDF file rather than as raw numerical data (1-D data) ^16 – 18^. The example of a PDF-based 2-D ECG is depicted in Figure 2. Due to this problem, researchers were required to convert the PDF file of the ECG into 1-D data before performing further emotion analysis, adding complexity to the pre-processing process. On this account, given the positive results obtained in monitoring and diagnosing cardiovascular-related diseases, the efficacy of 2-D ECG in emotion studies also warrants further investigation.”

**How was the data annotated in the DREAMER dataset? Were they continuous annotations or a single annotation per video clip? This is very relevant to include in the article. A few more words about the dataset are needed like a short description of the experimental protocol** **.**

 Thank you for bringing this issue to our attention. The short description of the DREAMER dataset has been improved to address this issue. The revised text was located in the Method section under a subsection called "The dataset (DREAMER") on page 8:

 “This study used ECG signals from Katsigiannis and Ramzan ^13^ called DREAMER. The DREAMER dataset is a freely accessible database of electroencephalogram (EEG) and electrocardiogram (ECG) signals used in emotion research. However, EEG signals were removed from this study because the primary focus is on ECG signals. The ECG was recorded using the SHIMMER ECG sensor at 256 Hz and stored in 1-D format. The DREAMER dataset contains 414 ECG recordings from 23 subjects who were exposed to 18 audio-visual stimuli designed to evoke emotion. Each participant assessed their emotions on a scale of 1 to 5 for arousal, valence, and dominance. However, because this study was primarily concerned with arousal and valence ratings, participants' evaluations of dominance were discarded.”

**Arousal/valence rating values are ranging from 1 to 5 in the DREAMER dataset. The authors never explain how they split them into two classes (high/low) for the binary classification** **.**

 Thank you for bringing this to our attention. We have improved this part by adding an explanation of how we categorise emotions into two classes. The explanation can be found on page 13:

 “The scale of self-assessed emotions, which ranges from 1 (lowest) to 5 (highest), was classified using a five-point scale with middle-point thresholds (an average rate of 3.8). As a result, scales four and five were assigned to the high class, while the remaining scales were assigned to the low class.”

**Authors do not include sufficient information on how the time-series ECG data was converted to an image (resolution, compression, windowing), or how the data was treated in general. Did the authors use the entire ECG signal for each video? Was there any windowing? Since the authors did not properly summarize the dataset (how were the videos annotated?) it is difficult to grasp or guess on how the data was processed.**

 We agree with this comment. Therefore, we improved the explanation part of pre-processing the 2-D ECG. The improvised text can be found in the first and second paragraphs of subsection 2-D ECG on page 11.

 “The duration of the ECG recording varies according to the duration of the video (average = 199 seconds). As Katsigiannis and Ramzan proposed, this study analysed the final 60 seconds of each recording to allow time for a dominant emotion to emerge ^13^. Following that, 1-D ECG was pre-processed using a simple MATLAB function by ^34^ to eliminate baseline wander caused by breathing, electrically charged electrodes, or muscle noise. The signal was then divided into four segments corresponding to 15 seconds each. Then, using MATLAB version 9.7, the 1-D ECG was transformed into a 2-D ECG (Figure 4). The image has a width of 1920 pixels and a height of 620 pixels.

 Due to the fact that the 2-D ECG was converted to a rectangle shape, it is not easy to resize the photos to the standard input image sizes of 224×224 and 299×299. As a result, the converted 2-D ECG was resized to 60% of its original size using Python version 3.8.5. This scale percentage was chosen after considering the quality of the image, the type of feature extractor used, and the computational cost the system can afford. The coloured images were converted into greyscale images. Then, binarization of the image using an Otsu's automatic image thresholding method ^35^ was done. This method ascertains the optimal threshold values from pixel values of 0 to 255 by calculating and evaluating their within-class variance ^36^”

**None of the cited literature used any of the image feature extraction methods that the authors used, and the authors did not discuss their reasoning for why they selected those image feature extraction methods and not the ones established in the ECG image analysis literature that they cited. Some more illustrations of these features may be useful besides the one in Figure 5 in order to convince the readers.**

 Thank you for pointing this out. Cite literature either using their own algorithm to detect peaks on PQRST waves or automatically extracting ECG features using a deep learning system. However, we had included our reason for employing these image feature extraction methods, which we believed could add a valuable contribution to the state-of-the-art. Additionally, we removed Figure 5 and replaced it with the description of each feature extractor to help convince the readers. The revised text can be found on pages 11 and 12:

 “The area of interest for 2-D ECG is laying on the PQRST waves, making the peaks detector the best approach to be employed. Therefore, six different feature extractors that could extract peaks, edges, or corners were applied to extract features from 2-D ECGs using Python version 3.8.5:

 1. ORB ^37^: ORB features are invariant to rotation and noise because they are a combination of Features from Accelerated Segment Test (FAST) detection and Binary Robust Independent Elementary Features (BRIEF) description methods.

 2. SIFT ^38^: SIFT identifies feature points by searching for local maxima on the images using Difference-of-Gaussians (DoG) operators. The description approach generates a 16x16 neighbourhood around each identified feature and sub-blocks the region. SIFT is also rotation and scale invariant.

 3. KAZE ^39^: KAZE is based on the scale of the normalised determinant of the Hessian Matrix, with the maxima of detector responses being captured as feature points using a moving window. Additionally, KAZE makes use of non-linear space via non-linear diffusion filtering to reduce noise while keeping the borders of regions in images.

 4. AKAZE ^40^: AKAZE is a more sophisticated version of KAZE that is based on the Hessian Matrix determinant. Scharr filters were employed to enhance the quality of the invariance rotation, rendering AKAZE features rotation- and scale-invariant.

 5. BRISK ^41^: While searching for maxima in the scale-space pyramid, BRISK detects corners using the AGAST algorithm and filters them using the FAST Corner Score. Additionally, the BRISK description is based on the recognised characteristic direction of each feature, which is necessary for rotation invariance.

 6. HOG ^42^: HOG is a feature descriptor that is used to compute the gradient value for each pixel. The image shape denoted the edge or gradient structure derived using a high-quality local gradient intensity distribution.”

**Support vector machine: It is not clear what preprocessing steps were applied to the data. For example, in the DREAMER dataset baseline they only use the last 60s of data for each film clip.**

 Thank you for this comment. The pre-processing part can be found in the Methods section under the 1-D ECG and 2-D ECG subsections. The pre-processing for 1-D ECG can be read as follows:

 “The AUBT and TEAP feature extractors were included with the Low Pass Filter (LPF), a filter meant to reject all undesirable frequencies in a signal. The LPF was one of the most widely used filters before the computation of statistical features for physiological signals ^31, 32^. As a result, automated 1-D ECG pre-processing utilizing LPF was performed in this study to reduce muscle and respiratory noise in ECG signals.”

 Whereas the pre-processing for 2-D ECG can be read as follows:

 “The duration of the ECG recording varies according to the duration of the video (average = 199 seconds). As Katsigiannis and Ramzan proposed, this study analysed the final 60 seconds of each recording to allow time for a dominant emotion to emerge ^13^. Following that, 1-D ECG was pre-processed using a simple MATLAB function by ^34^ to eliminate baseline wander caused by breathing, electrically charged electrodes, or muscle noise. The signal was then divided into four segments corresponding to 15 seconds each. Then, using MATLAB version 9.7, the 1-D ECG was transformed into a 2-D ECG.”

**Data was divided 80:20 and 10-fold cross-validation was used. The authors do not specify exactly how the data was split (see DREAMER dataset paper section V as an example). From reading this part, I can only assume that all data from all participants was used for each fold (general model), something that is diverging from how the data was split in the DREAMER dataset paper (they made models for each individual participant). Therefore, any comparisons to the results of the DREAMER baseline are invalid.**

 Thank you for pointing this out. We have addressed this issue by adding a new subsection under Method, namely, Experimental Setting. This subsection is located on pages 13 and 14, and can be read as follows:

 “The hyperparameters for SVM were tuned using an exhaustive parameter search tool, GridSearchCV, from Scikit-learn that automates the tuning procedure ^45^. This study tuned only the parameters with a high and relative tuning risk and left the remainder at their default values because they are the least sensitive to the hyperparameter tuning process, as suggested by Weerts, Mueller, and Vanschoren ^46^.

 The dataset was split into a reasonable proportion of training and testing sets to evaluate the model's performance on new unseen data. This study used a stratified train-test split of 80:20 for the training and testing sets. This strategy guarantees the dataset's exact proportions of samples in each class are preserved.

 Additionally, as we had a small dataset size, this study applied KFold Cross-Validation, with the number of folds set to 10, the most commonly used number in prior research to improve ERS performance.”

 Additionally, the summary of the experimental setting values has been tabulated in Table 5 on page 14.

**Results: If the classes are unbalanced (as the DREAMER dataset paper indicates) accuracy is not valid on its own, include f1 score, and/or Cohen's Kappa..**

 Thank you for your suggestions. As per your recommendations, we have included the F1-score along with the accuracy to address the unbalanced class distribution issue. This result can be found in Table 6 on page 14.

**Discussion: "Ref. 11" has nothing to do with the statement in that paragraph. LDA was actually applied in the DREAMER dataset paper and they reported that there were no significant differences in performance.**

 Thank you for your great observations. We have revised that part as needed. Regarding the LDA, we agree with you. However, as far as we know, LDA was used for classification purposes in the DREAMER dataset paper but not for dimension reduction.

# Peer review: Reviewer response for version 1

**DOI:** 10.5256/f1000research.76896.r121060

**Published:** 2022-02-21

**Authors:** Umesh Chandra Pati

**Affiliations:** 1 Department of Electronics and Communication Engineering, National Institute of Technology, Rourkela, Odisha, India

The paper presents a comparison of the Emotion Recognition System (ERS) performance using ECG image and ECG numerical data. DREAMER dataset containing 23 ECG recordings has been used for experimentation. ECG numerical data has been converted to ECG images for comparison. The proposed method consists of feature extraction, dimension reduction, and emotion classification. Arousal and Valence accuracy have been compared using different feature extractors on ECG image and numerical data. ORB, SIFT, KAZE, AKAZE, BRISK, and HOG have been used to extract features from ECG image data whereas TEAP and AUBT have been used to extract features from ECG numerical data. It has been concluded that ECG numerical data provides better performance of emotion classification in comparison to ECG image data.

 The authors have done a good job in addressing a typical issue. However, there are many aspects that need attention. Hence, the following suggestions should be addressed to enhance the quality of the manuscript.

1. Literature survey is poor. More number of related state-of-the-art works should be cited. There is no citation of the work taking into account both ECG image and ECG numerical data.
2. It has been mentioned that the converted ECG images have been resized to 60% of the original size to reduce the computational time. What is the reason for choosing 60%?
3. There is no analysis of the computational complexity of the proposed method.
4. In Table 4, emotion classification accuracies for both ECG image and ECG numerical data have been provided. Can these accuracy values be accepted in practical applications? 
5. There is no comparison of the proposed method with state-of-the-art methods.
6. References should be complete in all respect and in a uniform style. A few exceptions are 1, 32, 39, 40.

Is the work clearly and accurately presented and does it cite the current literature?

Partly

If applicable, is the statistical analysis and its interpretation appropriate?

Yes

Are all the source data underlying the results available to ensure full reproducibility?

Yes

Is the study design appropriate and is the work technically sound?

Yes

Are the conclusions drawn adequately supported by the results?

Yes

Are sufficient details of methods and analysis provided to allow replication by others?

Yes

Reviewer Expertise:

Computer Vision, Image/Video Processing, IoT, Artificial Intelligence.

I confirm that I have read this submission and believe that I have an appropriate level of expertise to confirm that it is of an acceptable scientific standard, however I have significant reservations, as outlined above.

# Response

**Published:** 2022-05-20

**Authors:** Sharifah Noor Masidayu Sayed Ismail

**The paper presents a comparison of the Emotion Recognition System (ERS) performance using ECG image and ECG numerical data. DREAMER dataset containing 23 ECG recordings has been used for experimentation. ECG numerical data has been converted to ECG images for comparison. The proposed method consists of feature extraction, dimension reduction, and emotion classification. Arousal and Valence accuracy have been compared using different feature extractors on ECG image and numerical data. ORB, SIFT, KAZE, AKAZE, BRISK, and HOG have been used to extract features from ECG image data whereas TEAP and AUBT have been used to extract features from ECG numerical data. It has been concluded that ECG numerical data provides better performance of emotion classification in comparison to ECG image data.**

**The authors have done a good job in addressing a typical issue. However, there are many aspects that need attention. Hence, the following suggestions should be addressed to enhance the quality of the manuscript.**

 Thank you for taking the time to review our manuscript. We reviewed the comments and made improvements based on the comments provided accordingly.

**Literature survey is poor. More number of related state-of-the-art works should be cited. There is no citation of the work taking into account both ECG image and ECG numerical data.**

 We have revised Section Related Works as per the suggestion by adding more related state-of-the-art work citations. The revised part is on pages 5 until 8. We also include the summary information about the existing works that employed 1-D and 2-D ECG input, which is tabulated in Table 2 on pages 6 through 8. Furthermore, the work that considers both ECG images and ECG numerical data is also included in this section. This work can be found on page 6, which reads as follows:

 "Additionally, Mandal et al. ^5^ published a study comparing 1-D and 2-D ECGs for the diagnosis of Ventricular Arrhythmia. They concluded that both ECG inputs are effective at detecting the disease."

**It has been mentioned that the converted ECG images have been resized to 60% of the original size to reduce the computational time. What is the reason for choosing 60%?**

 Thank you for drawing our attention to this. We have improved the explanation of this matter in the manuscript. You can find the text on page 11 that reads as follows:

 “Due to the fact that the 2-D ECG was converted to a rectangle shape, it is not easy to resize the photos to the standard input image sizes of 224×224 and 299×299. As a result, the converted 2-D ECG was resized to 60% of its original size using Python version 3.8.5. This scale percentage was chosen after considering the quality of the image, the type of feature extractor used, and the computational cost the system can afford.”

**There is no analysis of the computational complexity of the proposed method** **.**

 Thank you for pointing out this matter. We have included the analysis of the computational complexity under the result section, tabulated in Table 7, which discussed the time analysis of both inputs used to train and test the ERS model. The analysis was located on page 14: 

 “For comparison purposes, the computation time for both ECG inputs was recorded and reported in Table 7. The average time required to compute 1-D is 1.58 ± 0.07 seconds. In comparison, the average computation time for 2-D is 3377.425 ± 3138.875 seconds. Therefore, according to the observation, 2-D took the longest computation time, whereas 1-D obtained the shortest.”

 Additionally, the analysis of the computational time was touched on in the Discussion and Conclusion section as follows:

 “In terms of computational cost, 1-D ECG is better to 2-D ECG since it requires less computation time.”

**In Table 4, emotion classification accuracies for both ECG image and ECG numerical data have been provided. Can these accuracy values be accepted in practical applications?**

 Thank you for pointing this out. For your information, our results have been updated according to the result of the latest experiment. This result is tabulated in Table 6 on page 13. Based on this result, the accuracy and F1-score achieved for this study are on par with the existing works, which shows that our ERS model can be accepted in practical applications. However, this does not rule out the possibility of improving this result, as there is much more room to improve the ERS performance to develop a more robust ERS in the future.

**There is no comparison of the proposed method with state-of-the-art methods** **.**

 A comparison with existing work (DREAMER) has been provided for 1-D ECG. This comparison can be found in the result section on page 15, tabulated in Table 6. However, no existing work using the 2-D ECG of the DREAMER dataset has been reported. Therefore, no comparison can be made.

**References should be complete in all respect and in a uniform style. A few exceptions are 1, 32, 39, 40.**

 Thank you for bringing our attention to this. We have revised the references and citation part accordingly and completed it in all respects and in a uniform style as per suggestions.
